# A new approach for fast field calculation in electrostatic electron lens design and optimization

**DOI:** 10.1038/s41598-024-55518-3

**Published:** 2024-02-28

**Authors:** Neda Hesam Mahmoudi Nezhad, Mohamad Ghaffarian Niasar, Cornelis W. Hagen, Pieter Kruit

**Affiliations:** 1 Department of Imaging Physics, Delft University of Technology, Delft, The Netherlands; 2 Faculty of Electrical Engineering, DC Systems, Energy Conversion and Storage, Delft University of Technology, Delft, The Netherlands

**Keywords:** Design, synthesis and processing, Imaging techniques, Scanning electron microscopy, Applied physics, Electronics, photonics and device physics, Optical physics

## Abstract

In electron optics, calculation of the electric field plays a major role in all computations and simulations. Accurate field calculation methods such as the finite element method (FEM), boundary element method and finite difference method, have been used for years. However, such methods are computationally very expensive and make the computer simulation challenging or even infeasible when trying to apply automated design of electrostatic lens systems with many free parameters. Hence, for years, electron optics scientists have been searching for a fast and accurate method of field calculation to tackle the aforementioned problem in the design and optimization of electrostatic electron lens systems. This paper presents a novel method for fast electric field calculation in electrostatic electron lens systems with reasonably high accuracy to enable the electron-optical designers to design and optimize an electrostatic lens system with many free parameters in a reasonably short time. The essence of the method is to express the off-axis potential in an axially symmetrical coordinate system in terms of derivatives of the axial potential up to the fourth order, and equate this to the potential of the electrode at that axial position. Doing this for a limited number of axial positions, we get a set of equations that can be solved to obtain the axial potential, necessary for calculating the lens properties. We name this method the fourth-order electrode method because we take the axial derivatives up to the fourth order. To solve the equations, a quintic spline approximation of the axial potential is calculated by solving three sets of linear equations simultaneously. The sets of equations are extracted from the Laplace equation and the fundamental equations that describe a quintic spline. The accuracy and speed of this method is compared with other field calculation methods, such as the FEM and second order electrode method (SOEM). The new field calculation method is implemented in design/optimization of electrostatic lens systems by using a genetic algorithm based optimization program for electrostatic lens systems developed by the authors. The effectiveness of this new field calculation method in optimizing optical parameters of electrostatic lens systems is compared with FEM and SOEM and the results are presented. It should be noted that the formulation is derived for general axis symmetrical electrostatic electron lens systems, however the examples shown in this paper are with cylindrical electrodes due to the simplicity of the implementation in the software.

## Introduction

In electron optics, the quality of an electrostatic lens system is specified by its electron-optical properties, which are determined from its electric field^1–3^. Calculation of the electric field for electrostatic lens systems is hence the most significant step needed in evaluation of the lens system. Therefore, the speed and accuracy of calculating the electric field (or electric potential) plays a major role in electron-optics computation, design and optimization. The electric field can be calculated using numerical methods such as FEM^4^, Boundary Element Method (BEM)^5^ and Finite Difference Method (FDM)^6^. Although these methods result in very accurate values for the electric field (which consequently results in accurate determination of optical parameters), all these methods are time consuming. For instance, calculation of the potential distribution using FEM and performing ray tracing takes ~1 min for each system^7^. Note: All computer simulations in this study were conducted on our system, a PC equipped with an Intel® Xeon® W-2123 CPU @3.60 GHz and 32 GB of RAM, using the MATLAB programming language. The reported computation times are estimated based on the results obtained on the mentioned PC and programming language. However, as demonstrated in the following sections, the study primarily focuses on the comparative analysis of computational times across different methods, making the specific PC and programming language used less significant; what matters are the relative numbers.

Currently it is not difficult to find electron-optical software such as SIMION^8^, EOD^9^, GPT^10^, CPO^11^, etc. to design/optimize electron lens systems which conducts the field calculations by the aforementioned accurate field calculation methods. However, in the design process they only change one lens geometric parameter or the voltages to influence the aberrations or to auto-focus, they are not capable of changing the whole shape of the lens. Assuming these programs can be used in an optimization loop by changing all lens geometries and voltages as free parameters, it can take many days or even weeks to get the results due to the computational time required for the accurate field calculation^7,12^. For instance, the design of even a simple system using FEM by COMSOL (a multi-physics design modelling software^13^) takes such a long computational time that the designer might not have the patience to wait for the result^7,12^.

In recent years, a method is presented by Lars et al.^14^ for the optimization of ion lens design based on an accurate field calculation (FEM), by implementing an adjoint optimization. This works in a feasible short time and is suitable for multi-objective functions of up to two. However, in electrostatic lens design, we sometimes want more than two objective functions in the design process. Moreover, we have found that electrostatic lens design optimization often has multiple local minima^15^. This hence requires to implement a global optimization technique such as an evolutionary algorithm instead of a local optimization technique, such as the adjoint method (further insights into the evaluation of popular evolutionary optimization algorithms, including Genetic Algorithms (GA)^16,17^, Particle Swarm Optimization (PSO)^18^, and Simulated Annealing (SA)^19^, alongside a gradient-based local optimization^17^, for the optimization of electrostatic lens systems, along with their comparisons, can be found in our other recent study^20,21^). As a consequence, implementation of any of the above mentioned previously-existed accurate field calculation methods in electron lens system optimization programs (to find the optimum shape of electrodes and potentials), in which usually thousands of systems must be evaluated to obtain a reasonable result, makes the total optimization slow and impractical^7,12^. Therefore, a fast field calculation method is highly demanded in the field of charged particle optical lens design.

Adriaanse et al. proposed a fast method to calculate the electric field by means of approximation of the axial potential with cubic splines and solving a set of linear equation^22–24^. In their method, the terms of the Laplace equation with higher order than the second derivative are truncated and the method is named the Second-Order Electrode Method (SOEM). Hesam et al. compared the accuracy of SOEM and FEM methods as well as their computational time for optical parameter calculations in electrostatic lens systems^7,25^. It was seen that due to the approximation applied, SOEM although very fast (~ 0.4 s for each system evaluation), suffers from inaccuracy, specifically for the calculation of optical parameters, such as aberration coefficients. Hesam et al.^7,12^ proposed a fully-automated technique, utilising a combination of SOEM and FEM, by implementing the Genetic Algorithm (GA) for optimization of multi-electrostatic lens systems which made their design possible with all geometries and voltages of the lens system as free parameters in a reasonable time (around several hours for combination of SOEM and FEM, compared to many days if only FEM was used)^7,11^. The time-consuming aspect of the described method is associated with the 'fine-tuning' phase of FEM. During this stage, data is input into another segment of the program responsible for recalculating optical parameters using FEM. This iterative process refines and corrects results obtained from SOEM, thereby significantly enhancing the accuracy of optical parameter calculations. This fine-tuning is essential and necessary to ensure the reliability of the overall optimization.

In the previously presented approach based on SOEM, first running a GA-aided optimization based on field calculation by SOEM is executed. Subsequently, the optimized system’s data is used as the initial input for another GA-aided optimization based on field calculation by FEM and to run this part as another full-optimization. Due to the inaccuracies in the field calculation by SOEM, this fine-tuning process for SOEM involves a full-GA-aided optimization run with multiple iterations to correct the systems and identify optimized configurations, where their optical parameters are calculated with the high accuracy of FEM (referred to as SOEM + FEM + GA).

Hence, though it is major progress for electron optics designers to tackle the challenge of designing electrostatic lens systems in a fully-automated way in a reasonable time, the computational time is still not considered to be very short. This is due to the degree of optical parameter calculation inaccuracy in SOEM, which needs more time to be fine-tuned by FEM at the end of the optimization. To increase the accuracy of the field calculation part by SOEM, the Laplace equations therefore need to be solved by considering the higher order terms as well.

In this paper, a new method for fast and reasonably accurate field calculation is presented. This method is based on solving the Laplace equation by retaining terms up to the fourth order derivatives. Hence the method is named the Fourth-Order Electrode Method (FOEM). The accuracy and computation time for field calculation of this method is evaluated and compared with the existing methods SOEM and FEM.

Next, the recently introduced FOEM method is incorporated into an optimization routine utilizing a Genetic Algorithm to assess its efficiency in optimizing electrostatic lens systems, referred to as FOEM + GA and its comparison with a GA-aided optimization based on field calculation by FEM (FEM + GA). In this context, efficiency refers to the time and effort needed to discover an optimized lens system with accurately calculated optical parameters, considering the quality of the optimized lens system found by that method within the given time.

After evaluating the efficiency of FOEM + GA and FEM + GA methods, further analysis is crucial. The results of the FOEM + GA optimization are then employed in another optimization based on FEM calculations, referred to as FOEM + FEM + GA. This analysis aims to determine whether, for FOEM + GA to achieve optical parameter calculation accuracy equivalent to FEM, a comprehensive optimization run based on FEM (as the fine-tuning step) is necessary after GA + FOEM, or alternatively, the FOEM + GA alone is sufficiently effective, requiring only a brief fine-tuning step by FEM (potentially by just a few iterations in FOEM + FEM + GA) rather than a complete FOEM + FEM + GA optimization with an extensive number of iterations.

Additionally, a comparison with the previously introduced SOEM based optimization method (SOEM + FEM + GA^7,12^) is essential to investigate the superiority of the newly introduced method based on FOEM in optimizing electrostatic lenses.

This paper is organised as follows. In “Mathematical derivation of the axial potential for electrostatic electron lens systems using FOEM” section, the mathematical derivation for the axial potential calculation using FOEM is explained and presented. To derive the axial potential formulation, first the fundamental equations of the quintic spline^26^ for a dataset with unequal gaps between the data points are derived in “Fundamental equations of the quintic spline”. Next, in “Solving the Laplace equations up to the fourth order derivatives” section, using these equations in combination with the Laplace equation and proper boundary conditions, a system of linear equations is formed, from which the axial potential and its first and second derivatives are calculated. The accuracy and speed of the field and optical parameters calculation of this new method in comparison with FEM and SOEM is presented in “Accuracy of FOEM in field and optical parameters calculation” section for different typical electrostatic lens systems including 3, 4 and 5 lens electrodes. In “Optimization of electrostatic electron lens systems based on FOEM and a comparison with SOEM- and FEM-based optimization” section, the newly introduced field calculation method (FOEM) is implemented in a genetic algorithm-based optimization program (FOEM + GA).

Subsequently, in “The calculation of optical parameters in a single run of GA Optimization based on FOEM, versus SOEM and FEM” section, the improvement trends and accuracy calculations of optical parameters for the optimized systems in a single run of GA-aided optimization based on FOEM are investigated and compared with situations where the optimized systems are calculated using SOEM and FEM. This is done to obtain more statistically reliable results for the accuracy comparisons of optical parameters calculated using different methods of FOEM, FEM, and SOEM. Additionally, we illustrate and compare the trends of improvements in finding better lens systems in the mentioned single run of GA optimization based on FOEM with situations where the optimized systems are calculated using SOEM and FEM.

In “The efficiency of GA optimization based on FOEM, FEM and SOEM” section, a comparative study on the efficiency of various GA-aided optimization approaches based on FOEM, FEM, and SOEM is presented. Building on this study, to assess the efficiency of FOEM + GA, the results of FOEM + GA are fed into another optimization based on FEM (FOEM + FEM + GA), and the outcomes are presented at the end in “The efficiency of GA optimization based on FOEM, FEM and SOEM” section. Conclusions are drawn in “Conclusion” section.

Note: The formula derived and applicable for electrostatic electron lens systems in both cylindrical and non-cylindrical configurations. However, the examples showcased in this paper utilize cylindrical electrodes. This choice is driven by the software's ease of implementation.

## Mathematical derivation of the axial potential for electrostatic electron lens systems using FOEM 

**Figure 1:**
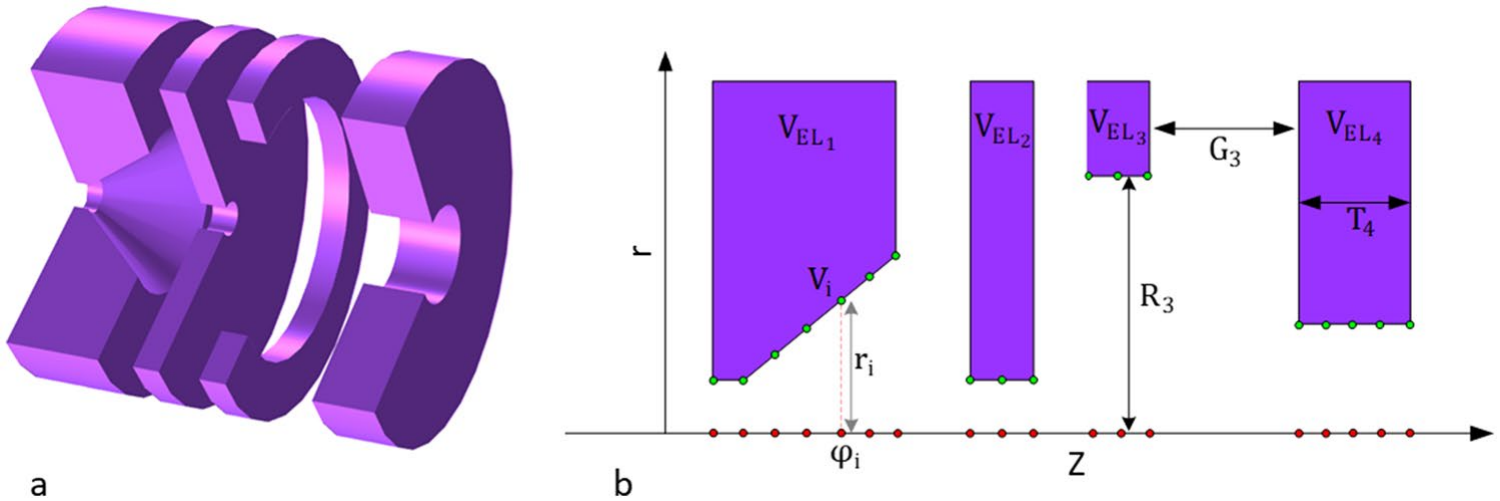
(**a**) Illustration of the rotationally symmetrical electrostatic lens system with 4 electrodes in a 3D representation. (**b**) Schematic depicting the rotationally symmetrical electrostatic lens system with 4 electrodes in a 2D representation. Here, $T_{j}$, $R_{j}$, and $V_{{EL_{j} }}$ denote the thicknesses, radii, and voltages at each electrode. $G_{j}$ represents the gaps between two consecutive electrodes.

**Figure 2:**
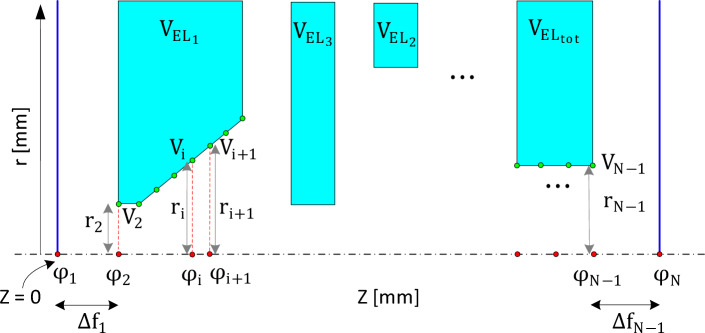
A typical rotationally symmetrical electrostatic lens system in 2D. The z-axis is the axis of rotational symmetry.

In this section, the aim is to derive the axial potential formulation for rotationally electrostatic lens systems with multi-electrodes. An illustrative example of such lens systems is provided schematically in both 3D (Fig. 1a) and 2D (Fig. 1b). Where, $T_{j}$, $R_{j}$, and $V_{{EL_{j} }}$ refer to the thicknesses, radii, and voltages at each electrode, respectively ($j = 1,2, \ldots ,tot$, where $tot$ is the total number of electrodes). While $G_{j}$ represents the gaps between two consecutive electrodes (($j = 1,2, \ldots ,tot - 1)$.

It can be demonstrated that for such geometries with axial symmetry, the spatial potential distribution can be obtained from the axial potential and its derivatives. The solution of the Laplace equation for a rotationally symmetrical geometry in terms of the axial potential and its derivatives is given by^27^

1 $$\varphi \left( {r,z} \right) = \varphi \left( {0,z} \right) - \frac{{r^{2} }}{4}\varphi^{\left( 2 \right)} \left( {0,z} \right) + \frac{{r^{4} }}{64}\varphi^{\left( 4 \right)} \left( {0,z} \right) + \cdots + \frac{{\left( { - 1} \right)^{m} r^{2m} }}{{\left( {m!} \right)^{2} 2^{2m} }}\varphi^{{\left( {2m} \right)}} \left( {0,z} \right) + \cdots$$

in which $r$ is the radial distance to the axis, $z$ is the horizontal distance from the entrance of the lens system, $\varphi \left( {r,z} \right)$ is the potential in space at coordinate $\left( {r,z} \right)$, $\varphi \left( {0,z} \right)$ is the axial potential at coordinate $\left( {0,z} \right)$, and $\varphi^{{\left( {2i} \right)}} \left( {0,z} \right)$ is the $\left( {2i} \right)$th derivative of the axial potential at coordinate $\left( {0,z} \right)$. When the symmetry axis is discretized into N points, and the terms are truncated after the fourth order derivative of the axial potential, Eq. (1) can be expressed as:

2 $$V_{i} \left( {r_{i} } \right) = \varphi_{i} - \frac{{r_{i}^{2} }}{4}\varphi_{i}^{\left( 2 \right)} + \frac{{r_{i}^{4} }}{64}\varphi_{i}^{\left( 4 \right)} \quad i = 2, 3, \ldots , N - 1$$

Considering Eq. (2), it is evident that the potential distribution along the axis ($\varphi_{i} )$ is linked to voltages at the surface of the electrodes ($V_{i} \left( {r_{i} } \right))$, where the values of $V_{i} \left( {r_{i} } \right))$ are known at each electrode and correspond to the electrode voltages assigned to that specific electrode ($V_{ELj} { }, j = 1,2, \ldots ,tot$).

However, as it is seen, directly deriving the voltage distribution along the axis ($\varphi_{i} )$ from this equation (i.e. Eq. (2)) is not feasible due to the greater number of unknowns compared to the available equations. Therefore, introducing additional conditions becomes necessary for solving the equations.

In this paper it is proposed that the axial potential can be effectively modelled by fitting it to a piecewise fifth-order spline equation. When combined with Laplace's equation, these equations can be solved together to determine the values of voltages along the axis.

### Fundamental equations of the quintic spline

While a method to construct the quintic spline for a dataset with equal intervals between data points is presented in^26^, it is important to note that, in general, a lens system may have unequal gaps between the electrodes and different thicknesses for the lenses. Consequently, the set of data points created on the electrodes may have unequal intervals. As there are no references found that derive the aforementioned equations for general unequal intervals, this section addresses that gap. Therefore, in the following section, the fundamental equations of the quintic spline are obtained and formulated for the scenario in which intervals between the data points are not equal.

To initiate the formulation, let's consider a dataset represented as $\left( {x_{i} ,\varphi_{i} } \right),$ where ${ }i = 1,2, \ldots ,{ }N$, comprising $N$ data points, each with two components. The first component is denoted as $x_{i}$, and its corresponding value is $\varphi_{i}$. Our goal is to fit splines to this set of $N$ data points. Specifically, a spline is fitted between data points $\left( {x_{i} ,\varphi_{i} } \right){ }$ and $\left( {x_{i + 1} ,\varphi_{i + 1} } \right).$

For a data set $\left\{ {\left( {x_{i} ,\varphi_{i} } \right)} \right\}, i = 1, 2, \ldots , N$, the quintic spline function $S\left( x \right)$ is defined by (3):

3 $$S\left( x \right) = S_{i} \left( x \right), \quad x \in \left[ {x_{i} , x_{i + 1} } \right], \;\;i = 1, 2, \ldots , N - 1$$

in which $S_{i} \left( x \right)$ is a fifth order polynomial defined on the interval $\left[ {x_{i} , x_{i + 1} } \right]$ and $S_{i} \left( {x_{i} } \right) = \varphi_{i}$.

To ensure a smooth transition from one spline to the next, additional conditions are imposed:

4 $$S_{i}^{\left( r \right)} \left( {x_{i + 1} } \right) = S_{i + 1}^{\left( r \right)} \left( {x_{i + 1} } \right),\quad r = 0,{ }1,{ }2,{ }3,{ }4{ }$$

where $S_{i}^{\left( r \right)} \left( x \right)$ is the $r$th derivative of $S_{i} \left( x \right)$.

When all these splines are combined, a generalized spline named S(x) is formed. At the data points on this generalized spline, certain conditions are imposed during spline construction, as mentioned above.

Since the polynomial is fifth order, the fourth derivative of the polynomial is a linear polynomial. Therefore the 4th derivative of $S_{i} \left( x \right)$ can be written as (5).

5 $$S_{i}^{\left( 4 \right)} \left( x \right) = Z_{i + 1} \frac{{\left( {x - x_{i} } \right)}}{{\Delta_{i} }} + Z_{i} \frac{{\left( {x_{i + 1} - x} \right)}}{{\Delta_{i} }}$$

in which $Z_{i} = S_{i}^{\left( 4 \right)} \left( {x_{i} } \right),\;\; x \in \left[ {x_{i} , x_{i + 1} } \right]$, and $\Delta_{i} = x_{i + 1} - x_{i}$. Integrating (5) four times, and after some rearrangements, Eq. (6) can be obtained.

6 $$S_{i} \left( x \right) = Z_{i + 1} \frac{{\left( {x - x_{i} } \right)^{5} }}{{120\Delta_{i} }} + Z_{i} \frac{{\left( {x_{i + 1} - x} \right)^{5} }}{{120\Delta_{i} }} + A_{i} \left( {x - x_{i} } \right)^{3} + B_{i} \left( {x_{i + 1} - x} \right)^{2} + C_{i} \left( {x - x_{i} } \right) + D_{i} \left( {x_{i + 1} - x} \right)$$

In (6), $A_{i} , B_{i} , C_{i} ,$ and $D_{i}$, $i = 1, 2, \ldots , N - 1$ are coefficients which can be determined in terms of $\varphi_{i} , \mu_{i} , \eta_{i} ,$ and $Z_{i}$, which are defined in (7).

7 $$\varphi_{i} = S_{i} \left( {x_{i} } \right) ,\;\; \mu_{i} = S_{i}^{\left( 2 \right)} \left( {x_{i} } \right) , \;\;\eta_{i} = S_{i}^{\left( 3 \right)} \left( {x_{i} } \right) ,\;\;Z_{i} = S_{i}^{\left( 4 \right)} \left( {x_{i} } \right)$$

Taking the derivative of (6) three times results in (8) and using the identities given by (7), results in (9), after simplification.

8 $$S_{i}^{\left( 3 \right)} \left( x \right) = Z_{i + 1} \frac{{\left( {x - x_{i} } \right)^{2} }}{{2\Delta_{i} }} - Z_{i} \frac{{\left( {x_{i + 1} - x} \right)^{2} }}{{2\Delta_{i} }} + 6A_{i}$$

9 $$A_{i} = \frac{{\Delta_{i} }}{12}Z_{i} + \frac{{\eta_{i} }}{6}$$

Equation (10) is then obtained by taking the derivative of Eq. (6) twice, which produces Eq. (11), using the identities given by Eq. (7) and upon simplification.

10 $$S_{i}^{\left( 2 \right)} \left( x \right) = Z_{i + 1} \frac{{\left( {x - x_{i} } \right)^{3} }}{{6\Delta_{i} }} + Z_{i} \frac{{\left( {x_{i + 1} - x} \right)^{3} }}{{6\Delta_{i} }} + 6A_{i} \left( {x - x_{i} } \right) + 2B_{i}$$

11 $$B_{i} = \frac{{\mu_{i} }}{2} - \frac{{\Delta_{i}^{2} }}{12}Z_{i}$$

Replacing $x = x_{i + 1}$ in (6) and using the identities given by (7) yields (12) which produces (13), after simplification.

12 $$\varphi_{i + 1} = Z_{i + 1} \frac{{\Delta_{i}^{4} }}{120} + A_{i} \Delta_{i}^{3} + C_{i} \Delta_{i}$$

13 $$C_{i} = \frac{{\varphi_{i + 1} }}{{\Delta_{i} }} - \frac{{\Delta_{i}^{3} }}{12}Z_{i} - \frac{{\Delta_{i}^{3} }}{120}Z_{i + 1} - \frac{{\Delta_{i}^{2} }}{6}\eta_{i}$$

Equation (14) is obtained by replacing $x = x_{i}$ in (6) and using the identities given by (7). Following simplification, using (11) in (14) then produces (15).

14 $$D_{i} = \frac{{\varphi_{i} }}{{\Delta_{i} }} - Z_{i} \frac{{\Delta_{i}^{3} }}{120} - B_{i} \Delta_{i}$$

15 $$D_{i} = \frac{{\varphi_{i} }}{{\Delta_{i} }} + \frac{{9\Delta_{i}^{3} }}{120}Z_{i} - \frac{{\mu_{i} }}{2}\Delta_{i}$$

In the equations below, the conditions of continuity of derivatives given in (4) for $r = 1, 2, 3$ are applied. For the case when $r = 3$, after simple evaluation, (16) and (17) are obtained. By equating (16) and (17) and using (9), Eq. (18) is derived.

16 $$S_{i}^{\left( 3 \right)} \left( {x_{i + 1} } \right) = Z_{i + 1} \frac{{\Delta_{i} }}{2} + 6A_{i}$$

17 $$S_{i + 1}^{\left( 3 \right)} \left( {x_{i + 1} } \right) = - Z_{i + 1} \frac{{\Delta_{i + 1} }}{2} + 6A_{i + 1}$$

18 $$\frac{{\Delta_{i} }}{2}Z_{i + 1} + \frac{{\Delta_{i} }}{2}Z_{i} + \eta_{i} - \eta_{i + 1} = 0$$

For the case when $r = 2,$ through a straightforward assessment, (19) and (20) are obtained. By equating (19) and (20) and using (9) and (11), Eq. (21) is produced.

19 $$S_{i}^{\left( 2 \right)} \left( {x_{i + 1} } \right) = Z_{i + 1} \frac{{\Delta_{i}^{2} }}{6} + 6A_{i} \Delta_{i} + 2B_{i}$$

20 $$S_{i + 1}^{\left( 2 \right)} \left( {x_{i + 1} } \right) = Z_{i + 1} \frac{{\Delta_{i + 1}^{2} }}{6} + 2B_{i + 1}$$

21 $$\eta_{i} = - \frac{{2\Delta_{i} }}{6}Z_{i} - \frac{{\Delta_{i} }}{6}Z_{i + 1} - \frac{{\mu_{i} }}{{\Delta_{i} }} + \frac{{\mu_{i + 1} }}{{\Delta_{i} }}$$

For the case when $r = 1$ and after simple evaluation, (22) and (23) are obtained. By equating (22) and (23) and using (9), (11), (13), and (15), Eq. (24) then results.

22 $$S_{i}^{\left( 1 \right)} \left( {x_{i + 1} } \right) = Z_{i + 1} \frac{{\Delta_{i}^{3} }}{24} + 3A_{i} \Delta_{i}^{2} + C_{i} - D_{i}$$

23 $$S_{i + 1}^{\left( 1 \right)} \left( x \right) = - Z_{i + 1} \frac{{\Delta_{i + 1}^{3} }}{24} - 2B_{i + 1} \Delta_{i + 1} + C_{i + 1} - D_{i + 1}$$

24 $$\frac{{11\Delta_{i}^{3} }}{120}Z_{i} + \frac{{4\Delta_{i}^{3} + 4\Delta_{i + 1}^{3} }}{120}Z_{i + 1} + \frac{{\Delta_{i + 1}^{3} }}{120}Z_{i + 2} + \frac{{2\Delta_{i}^{2} }}{6}\eta_{i} + \frac{{\Delta_{i + 1}^{2} }}{6}\eta_{i + 1} + \frac{{\Delta_{i} }}{2}\mu_{i} + \frac{{\Delta_{i + 1} }}{2}\mu_{i + 1} = \frac{{\varphi_{i + 2} }}{{\Delta_{i + 1} }} - \frac{{\varphi_{i + 1} }}{{\Delta_{i + 1} }} - \frac{{\varphi_{i + 1} }}{{\Delta_{i} }} + \frac{{\varphi_{i} }}{{\Delta_{i} }}$$

On substituting (21) in (18) and (24), Eqs. (25) and (26) are derived, which are referred to in this paper as the fundamental equations of the quintic spline.

25 $$\frac{{\Delta_{i} }}{6}Z_{i} + \frac{{2(\Delta_{i} + \Delta_{i + 1} )}}{6}Z_{i + 1} + \frac{{\Delta_{i + 1} }}{6}Z_{i + 2} - \frac{{\mu_{i} }}{{\Delta_{i} }} + \frac{{\mu_{i + 1} }}{{\Delta_{i} }} + \frac{{\mu_{i + 1} }}{{\Delta_{i + 1} }} - \frac{{\mu_{i + 2} }}{{\Delta_{i + 1} }} = 0\quad i = 1, 2, \ldots , N - 2$$

26 $$- \frac{{7\Delta_{i}^{3} }}{360}Z_{i} - \frac{{8\left( {\Delta_{i}^{3} + \Delta_{i + 1}^{3} } \right)}}{360}Z_{i + 1} - \frac{{7\Delta_{i + 1}^{3} }}{360}Z_{i + 2} + \frac{{\Delta_{i} }}{6}\mu_{i} + \frac{{2\left( {\Delta_{i} + \Delta_{i + 1} } \right)}}{6}\mu_{i + 1} + \frac{{\Delta_{i + 1} }}{6}\mu_{i + 2} = \frac{{\varphi_{i + 2} }}{{\Delta_{i + 1} }} - \frac{{\varphi_{i + 1} }}{{\Delta_{i + 1} }} - \frac{{\varphi_{i + 1} }}{{\Delta_{i} }} + \frac{{\varphi_{i} }}{{\Delta_{i} }} \quad i = 1, 2, \ldots , N - 2$$

### Solving the Laplace equations up to the fourth order derivatives

Once the quintic spline is formulated, the axial potential has to be numerically written up to the fourth order, to be simultaneously solved together with the spline equations.

A typical rotationally symmetrical electrostatic lens system in 2D is shown in Fig. 2. For a clearer visualization of the lens geometry in 2D and 3D, along with detailed explanations of its parameters, please refer to Fig. 1. The geometry of the electrodes, including the gaps between them and the voltages at each electrode, determine the lens properties.

As aforementioned, by omitting the terms above the fourth derivatives and using the identities in (27), Eq. (1) can be written numerically as (28).

27 $$\mu_{i} = \varphi_{i}^{\left( 2 \right)} \left( {0,z} \right) ,\;\;Z_{i} = \varphi_{i}^{\left( 4 \right)} \left( {0,z} \right)$$

28 $$V_{i} \left( {r_{i} } \right) = \varphi_{i} - \frac{{r_{i}^{2} }}{4}\mu_{i} + \frac{{r_{i}^{4} }}{64}Z_{i} \quad i = 2, 3, \ldots , N - 1$$

In electrostatic lens systems, it can be assumed that the axial potential before the first electrode and after the last electrode approaches the potential of the first and the final electrode, respectively. However, the exact position for which the above condition is valid must be identified. Referring to Fig. 2, this means the distances $\Delta f_{1}$ and $\Delta f_{N - 1}$ must be properly calculated. Equations (25) and (26) and (28) each form $N - 2$ equations, resulting in a total of $3N - 6$ equations, while there are $N$ unknown $\varphi_{i}$, $\mu_{i} , Z_{i}$ for $i = 1, 2, \ldots ,N$ which results in $3N$ unknowns in total. However, as stated above, it is assumed that $\varphi_{1} = V_{2}$ and $\varphi_{N} = V_{N - 1}$, in which $V_{2}$ is the voltage of the first electrode (i.e. $V_{EL1}$) and $V_{N - 1}$ is the voltage of the last electrode (i.e. $V_{EL\,tot}$), are both known values. Therefore, based on the above assumption, the two unknown parameters are already known, but instead, the distances $\Delta f_{1}$ and $\Delta f_{N - 1}$ are unknown, which again results in a total of $3N$ unknown parameters. Considering that there are only $3N - 6$ equations, this means 6 boundary conditions must be selected first. Here we assume $\mu_{1} = \mu_{N} = \eta_{1} = \eta_{N} = Z_{1} = Z_{N} = 0$. These assumptions are all reasonable, since before the first point and after the last point there are field-free regions and therefore assuming the second, third and fourth derivatives of voltage to be zero is also justifiable. Note that all voltages $V_{i}$ belonging to the same electrode j are equal to $V_{ELj}$.

It should also be noted that, In Eq. (28), we discretized the Z axis, and as a result, the Z coordinate is embedded in the indices of the parameters. For instance, z = 0 corresponds to $\varphi_{1}$, and with a discretization step of, for example, 0.001 mm, the point z = 0.001 is associated with $\varphi_{2}$, and so on. When we refer to the 'first electrode,' it implies that there are no other electrodes before it. Consequently, the voltage preceding the first electrode will be dominated by the voltage of the first electrode. This holds true for the last electrode as well. If any other part of the microscope preceding the first electrode has a voltage different from that of the first electrode, we assume that part to be the first electrode. Preceding this assumed first electrode, the voltage will tend to become equal to that of the first electrode.

### Calculation of $\Delta {\varvec{f}}_{1}$ and $\Delta {\varvec{f}}_{{{\varvec{N}} - 1}}$

**Figure 3:**
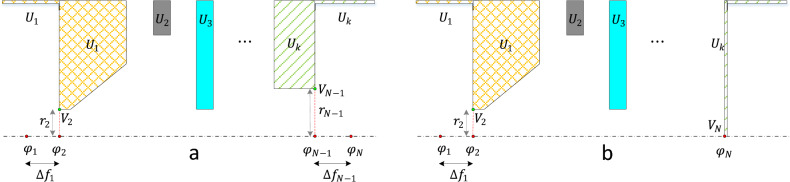
The illustration of the lens system depicts voltages at the outer radius of the lens, with the voltages before the first electrode and after the last electrode set to the voltage of the first electrode and the last electrode, respectively. In (**a**), there are holes in both the first and the last electrodes. In (**b**), there is a hole in the first electrode, while the last electrode is closed.

In this section, first the values of $\Delta f_{1}$ and $\Delta f_{N - 1}$ are calculated for the case when there is a hole (opening in the electrode near the axis) in the first and in the last electrodes, as shown in Fig. 3a. In this case it is assumed that the voltages at the outer radius of the lens before the first electrode and after the last electrode are set to the voltage of the first electrode and the last electrode, respectively. It means that on the axis, starting from the entrance of the lens toward the left (with reference to Fig. 3a), the voltage gradually approaches the voltage of the first electrode, and at point 1 the voltage is exactly the same as that of the first electrode. Based on these conditions it can be concluded that the electric field at the first and the last point is equal to zero.

The Taylor expansion of the axial potential for $\varphi_{2}$ and assuming $\Delta f_{1} = \Delta f$ can be written as:

29 $$\varphi_{2} = \varphi_{1} + \varphi_{1}{\prime} \frac{\Delta f}{{1!}} + \varphi_{1}^{^{\prime\prime}} \frac{{\Delta f^{2} }}{2!} + \varphi_{1}^{\left( 3 \right)} \frac{{\Delta f^{3} }}{3!} + \varphi_{1}^{\left( 4 \right)} \frac{{\Delta f^{4} }}{4!} + \varphi_{1}^{\left( 5 \right)} \frac{{\Delta f^{5} }}{5!} + \cdots$$

Considering the boundary conditions, and also the fact that the electric field at point 1 is zero, Eq. (29) can be simplified to (30) by ignoring terms with higher than fifth order derivatives. Alternatively, Eq. (31) can also be written. Combining (30) and (31) results in (32).

30 $$\varphi_{2} = \varphi_{1} + \varphi_{1}^{\left( 5 \right)} \frac{{\Delta f^{5} }}{5!}$$

31 $$\varphi_{1}^{\left( 5 \right)} = \frac{{\varphi_{2}^{\left( 4 \right)} - \varphi_{1}^{\left( 4 \right)} }}{\Delta f} = \frac{{\varphi_{2}^{\left( 4 \right)} }}{\Delta f}$$

32 $$\varphi_{2} = \varphi_{1} + \varphi_{2}^{\left( 4 \right)} \frac{{\Delta f^{4} }}{5!}$$

Considering Eq. (28) for $i = 2$, Eq. (33) can be obtained.

33 $$V_{2} = \varphi_{2} - \frac{{r_{2}^{2} }}{4}\varphi_{2}^{\left( 2 \right)} + \frac{{r_{2}^{4} }}{64}\varphi_{2}^{\left( 4 \right)}$$

Equation (21) for $i = 1$, by considering that in this case $\Delta_{1} = \Delta f$ and using the boundary conditions at point 1, can be written as Eq. (34):

34 $$\varphi_{2}^{\left( 2 \right)} = \frac{{\left( {\Delta f} \right)^{2} }}{6}\varphi_{2}^{\left( 4 \right)}$$

By combining Eqs. (33) and (34) and defining $X = \left( {\frac{{r_{2}^{4} }}{64} - \frac{{r_{2}^{2} }}{4}\frac{{\left( {\Delta f} \right)^{2} }}{6}} \right)$, Eq. (35) is derived:

35 $$\varphi_{2}^{\left( 4 \right)} = \frac{{V_{2} - \varphi_{2} }}{X}$$

By combining (32) and (35), Eq. (36) is obtained. Equating $\frac{{\Delta f^{4} }}{5!X} = - 1$ produces the desired result of $\varphi_{1} = V_{2}$, and results in Eq. (37), after some simplification .

36 $$\varphi_{1} = \varphi_{2} \left( {1 + \frac{{\Delta f^{4} }}{5!X}} \right) - V_{2} \frac{{\Delta f^{4} }}{5!X}$$

37 $$\Delta f^{4} - 5r_{2}^{2} \Delta f^{2} + \frac{15}{8}r_{2}^{4} = 0$$

**Figure 4:**
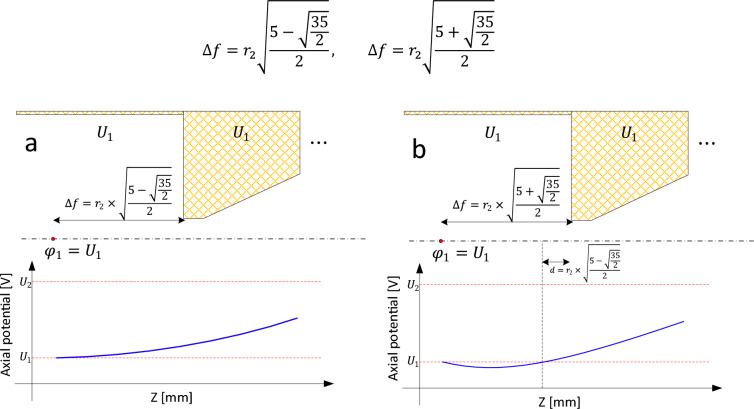
Proper selection of $\Delta f_{1}$ and $\Delta f_{N - 1}$ from the roots of (38). (**a**) axial potential smoothly approaches the voltage of the first electrode when $\Delta f$ is equal to the smaller root of (38), (**b**) axial potential oscillates before it reaches the voltage of the first electrode when $\Delta f$ is equal to the larger root of (38).

Equation (37) has four roots, two of which are negative and two positive (Eq. 38). The negative ones cannot be acceptable. From the two positive roots, only the smaller one (indicated in Fig. 4a) is acceptable. This is because if the larger one is selected as $\Delta f_{1 }$ (as shown in Fig. 4b), considering the larger root as point 1, the smaller root falls between point 2 and point 1. In this way, there is another point on the axis that has a potential equal to $V_{2}$. This means that the voltage before point 2 is not smoothly approaching $V_{2}$, but oscillating, as shown in Fig. 4b, which is not possible. Therefore, the larger root cannot be an acceptable solution of the equation.

38 $$\Delta f = r_{2} \sqrt {\frac{{5 - \sqrt{\frac{35}{2}} }}{2}} , \;\;\Delta f = r_{2} \sqrt {\frac{{5 + \sqrt{\frac{35}{2}} }}{2}}$$

The same concept is valid for the last electrode and $\Delta f_{N - 1}$ can be obtained. In this case, to obtain (34), Eqs. (18) and (21) must be evaluated for $i = N - 1$, and after combining them (34) is obtained. The rest of the procedure is the same and the smaller root shown in (38) can be used to calculate $\Delta f_{N - 1}$ with replacement of $r_{2}$ with $r_{N - 1}$.

It is worth mentioning that, on the boundary conditions, if the first electrode is open (contains a hole) and the last electrode is completely closed (no hole), as shown in Fig. 3b, the final data point is $\varphi_{N}$, and its voltage is known and is equal to $V_{N}$, the voltage of the last electrode. As before, there are $3N - 6$ equations, while there are $3N$ unknowns in total. However, $\varphi_{1} = V_{2}$ and $\varphi_{N} = V_{N}$, which means 2 unknowns are already known, but instead, the distance $\Delta f_{1}$ must be identified, which can be found using (38). For this case it is not necessary to add another point after the last electrode and, hence, $\Delta f$ does not exist after the last electrode. Therefore, only 5 extra boundary conditions are needed. The extra boundary conditions imposed are $\mu_{1} = \mu_{N} = \eta_{1} = Z_{1} = Z_{N} = 0$. Note that for this case it is not necessary to set $\eta_{N} = 0$. In this case, while the electric field at the first point is zero, the electric field at point $N$ is an unknown value which depends on the lens system’s geometry and voltages.

Note: The term 'hole,' as mentioned above, refers to the opening in the electrode near the axis. All electrodes must include an opening around the axis to facilitate the passage and flow of electrons, as depicted in Fig. 3a. However, the last electrode corresponds to the location of the image plane (sample plane), where it is generally closed (as shown in Fig. 3a). In some situations, it can be closed without the necessity for an opening (as shown in Fig. 3b). While such situations are infrequent, they may be of interest to electron lens designers for specific applications and therefore has been studied here as well. For a detailed exploration of these situations and their applications, refer to^12,28–30^.

### Matrix formulation of the equations 

First, the matrix formulations of the equations are explained for the scenario in which both the first and the last lenses have a hole in them. Assume $\left[ \varphi \right]_{N \times 1}$ is the vector of the axial potential, $\left[ \mu \right]_{N \times 1}$ is the vector of the second derivative of the axial potential, and $\left[ Z \right]_{N \times 1}$ is the vector of the fourth derivative of the axial potential. Equation (25) in the matrix form can be written as (39). Elements of matrices $\left[ A \right]$ and $\left[ B \right]$ can be obtained from (25). The close format of matrices are represented in the text. The open-format of matrices are given in the appendix for more clarity.

39 $$\left[ A \right]_{{\left( {N - 2} \right) \times N}} \left[ Z \right]_{N \times 1} = \left[ B \right]_{{\left( {N - 2} \right) \times N}} \left[ \mu \right]_{N \times 1}$$

With the boundary conditions $Z_{1} = Z_{N} = \mu_{1} = \mu_{N} = 0$, it is possible to reduce Eq. (39) and obtain (40). In Eq. (40) $\left[ {Z^{*} } \right]$ and $\left[ {\mu^{*} } \right]$ are the vectors of the fourth derivative and second derivative of axial potential, respectively, starting from point 2 until point $N - 1$. Matrices $\left[ {A^{*} } \right]$ and $\left[ {B^{*} } \right]$ are the reduced $\left[ A \right]$ and $\left[ B \right]$.

40 $$\left[ {A^{*} } \right]_{{\left( {N - 2} \right) \times \left( {N - 2} \right)}} \left[ {Z^{*} } \right]_{{\left( {N - 2} \right) \times 1}} = \left[ {B^{*} } \right]_{{\left( {N - 2} \right) \times \left( {N - 2} \right)}} \left[ {\mu^{*} } \right]_{{\left( {N - 2} \right) \times 1}}$$

Equation (26) in the matrix form can be written as (41). Elements of the matrices $\left[ C \right]$, $\left[ D \right]$ and $\left[ K \right]$ can be obtained from (26). With the boundary conditions $Z_{1} = Z_{N} = \mu_{1} = \mu_{N} = 0$, it is possible to reduce (41) to Eq. (42). In Eq. (42) matrices $\left[ {C^{*} } \right]$, $\left[ {D^{*} } \right]$ and $\left[ {K^{*} } \right]$ are the reduced $\left[ C \right]$, $\left[ D \right]$ and $\left[ K \right]$. In (42) $\left[ {U_{0} } \right]$ is a vector of size $\left( {N - 2} \right) \times 1$, in which element 1 is $\varphi_{1}$, which is equal to the voltage of the first electrode, element N is $\varphi_{N}$, which is equal to the voltage of the last electrode, and all the other elements are zero.

41 $$\left[ C \right]_{{\left( {N - 2} \right) \times N}} \left[ Z \right]_{N \times 1} + \left[ D \right]_{{\left( {N - 2} \right) \times N}} \left[ \mu \right]_{N \times 1} = \left[ K \right]_{{\left( {N - 2} \right) \times N}} \left[ \varphi \right]_{N \times 1}$$

42 $$\left[ {C^{*} } \right]_{{\left( {N - 2} \right) \times \left( {N - 2} \right)}} \left[ {Z^{*} } \right]_{{\left( {N - 2} \right) \times 1}} + \left[ {D^{*} } \right]_{{\left( {N - 2} \right) \times \left( {N - 2} \right)}} \left[ {\mu^{*} } \right]_{{\left( {N - 2} \right) \times 1}} = \left[ {K^{*} } \right]_{{\left( {N - 2} \right) \times \left( {N - 2} \right)}} \left[ {\varphi^{*} } \right]_{{\left( {N - 2} \right) \times 1}} + \left[ {U_{0} } \right]_{{\left( {N - 2} \right) \times 1}}$$

Equation (28) can be written in matrix form, as in (43). In Eq. (43), vector $\left[ V \right]$ represents the voltages of all points on the electrodes (therefore it is a known vector) and matrix $\left[ r \right]$ is a diagonal matrix of size $\left( {N - 2} \right) \times \left( {N - 2} \right)$ and the diagonal elements are the distances between each point on the electrode to its mapped counter point on the axis of symmetry.

43 $$\left[ V \right]_{{\left( {N - 2} \right) \times 1}} = \left[ {\varphi^{*} } \right]_{{\left( {N - 2} \right) \times 1}} - \frac{{\left[ r \right]_{{\left( {N - 2} \right) \times \left( {N - 2} \right)}}^{2} }}{4}\left[ {\mu^{*} } \right]_{{\left( {N - 2} \right) \times 1}} + \frac{{\left[ r \right]_{{\left( {N - 2} \right) \times \left( {N - 2} \right)}}^{4} }}{64}\left[ {Z^{*} } \right]_{{\left( {N - 2} \right) \times 1}}$$

Equation (40) can be written as (44). Using (44) and (42), Eq. (45) can be calculated. Combining Eq. (45) and (43) results in Eq. (46), after simplification. Rewriting (46), Eq. (47) can be obtained from which $\left[ {\varphi^{*} } \right]$ can be calculated. Using $\left[ {\varphi^{*} } \right]$ in (45) $\left[ {Z^{*} } \right]$ is derived and using $\left[ {Z^{*} } \right]$ in (44), $\left[ {\mu^{*} } \right]$ can be obtained. Adding the first and last element to the vectors $\left[ {\varphi^{*} } \right]$, $\left[ {\mu^{*} } \right]$ and $\left[ {Z^{*} } \right]$ using the boundary conditions, $Z_{1} = Z_{N} = \mu_{1} = \mu_{N} = 0$ and the fact that $\varphi_{1}$ is equal to the voltage of the first electrode and $\varphi_{N}$ is equal to the voltage of the last electrode, the full vectors of $\left[ \varphi \right]$, $\left[ \mu \right]$ and $\left[ Z \right]$ can be determined.

44 $$\left[ {\mu^{*} } \right] = \left[ {B^{*} } \right]^{ - 1} \left[ {A^{*} } \right]\left[ {Z^{*} } \right]$$

45 $$\left[ {Z^{*} } \right] = \left\{ {\left[ C \right] + \left[ D \right]\left[ {B^{*} } \right]^{ - 1} \left[ {A^{*} } \right]} \right\}^{ - 1} \left( {\left[ {K^{*} } \right]\left[ {\varphi^{*} } \right] + \left[ {U_{0} } \right]} \right)$$

46 $$\begin{aligned} & \left[ V \right] - \left( {\frac{{\left[ r \right]^{4} }}{64} - \frac{{\left[ r \right]^{2} }}{4}\left[ {B^{*} } \right]^{ - 1} \left[ {A^{*} } \right]} \right)\left\{ {\left[ C \right] + \left[ D \right]\left[ {B^{*} } \right]^{ - 1} \left[ {A^{*} } \right]} \right\}^{ - 1} \left[ {U_{0} } \right] \\ & \quad = \left( {I + \left( {\frac{{\left[ r \right]^{4} }}{64} - \frac{{\left[ r \right]^{2} }}{4}\left[ {B^{*} } \right]^{ - 1} \left[ {A^{*} } \right]} \right)\left\{ {\left[ C \right] + \left[ D \right]\left[ {B^{*} } \right]^{ - 1} \left[ {A^{*} } \right]} \right\}^{ - 1} \left[ {K^{*} } \right]} \right)\left[ {\varphi^{*} } \right] \\ \end{aligned}$$

47 $$\begin{aligned} & \left[ {\varphi^{*} } \right] = \left[ P \right]^{ - 1} \left[ Q \right] \\ & \left[ P \right] = \left( {I + \left( {\frac{{\left[ r \right]^{4} }}{64} - \frac{{\left[ r \right]^{2} }}{4}\left[ {B^{*} } \right]^{ - 1} \left[ {A^{*} } \right]} \right)\left\{ {\left[ C \right] + \left[ D \right]\left[ {B^{*} } \right]^{ - 1} \left[ {A^{*} } \right]} \right\}^{ - 1} \left[ {K^{*} } \right]} \right) \\ & \left[ Q \right] = \left[ V \right] - \left( {\frac{{\left[ r \right]^{4} }}{64} - \frac{{\left[ r \right]^{2} }}{4}\left[ {B^{*} } \right]^{ - 1} \left[ {A^{*} } \right]} \right)\left\{ {\left[ C \right] + \left[ D \right]\left[ {B^{*} } \right]^{ - 1} \left[ {A^{*} } \right]} \right\}^{ - 1} \left[ {U_{0} } \right] \\ \end{aligned}$$

Having established $\left[ \varphi \right]$, $\left[ \mu \right]$ and $\left[ Z \right]$, it is possible to formulate quintic spline equations $S_{i} \left( x \right)$ for $i = 1, 2, \cdots , N - 1$. $S_{i} \left( x \right)$ can be calculated using (6). Coefficients $A_{i}$, $B_{i}$, and $D_{i}$ can be directly calculated using (9), (11) and (15). To calculate $C_{i}$, $\eta_{i}$ for $i = 1, 2, \cdots , N - 1$ must first be determined from (21), and based on the assumed boundary condition $\eta_{N} = 0$. Having all $\eta_{i}$ and using (13), it is now possible to calculate $C_{i}$. If the last electrode is closed, the same strategy as explained above can be used to calculate $S_{i} \left( x \right)$ for $i = 1, 2, \cdots , N - 1$. The only difference is that, after determining $\eta_{i}$ for $i = 1, 2, \cdots , N - 1$, the value of $\eta_{N}$ should be calculated using (17) by setting $i = N - 1$.

Note: To solve the matrix equations, various software and programming languages like MATLAB or Python can be employed for this task. In general, a linear matrix equation in the form of $Ax = B$ can be easily solved, for example, in MATLAB by defining the matrices A and B and using the command $x = A^{ - 1} B$. Since all matrices presented in this Section can be computed from the geometry data and electrode voltages, the final matrices can be effortlessly calculated through a straightforward implementation in MATLAB. Following this, as explained, it is feasible to compute cubic splines, and from there, the voltage distribution along the axis of symmetry can be derived.

It is noted that for comparison with the here presented FOEM method, accurate field calculations were done using COMSOL Multiphysics, a FEM-based software tool. For mathematical formulations of solving the Poisson equation using FDM or FEM, the interested reader is referred to the literature^31–33^.

## Accuracy of FOEM in field and optical parameters calculation

**Figure 5:**
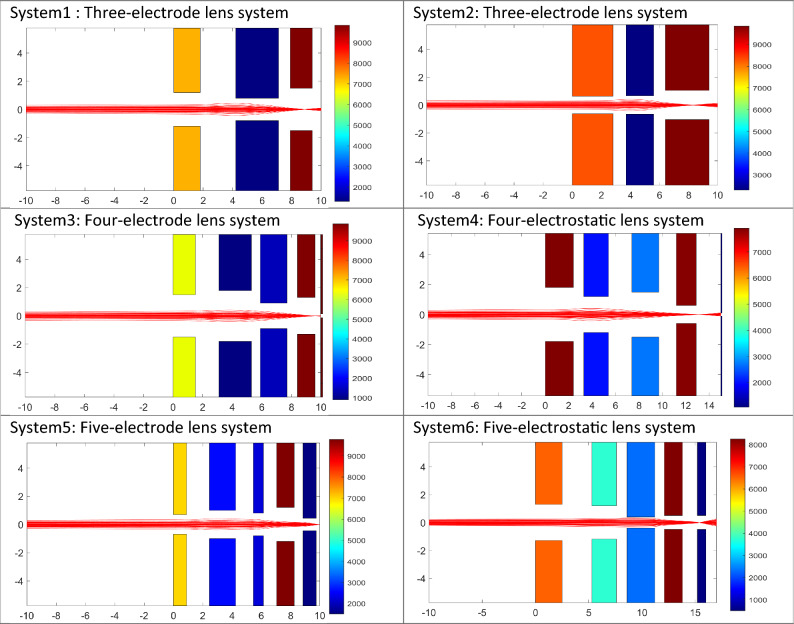
Six different typical electrostatic lens systems (in 2D) taken as test systems for making the comparison of the methods of SOEM, FOEM and FEM. The colours indicate the voltages at each electrode. The primary beam passing through the lens system (in red, as taken from our raytracing codes in MATLAB using FEM field calculation) is sketched for a better visualisation of the lens system. The units along the axes of the graphs are in millimetres.

**Figure 6:**
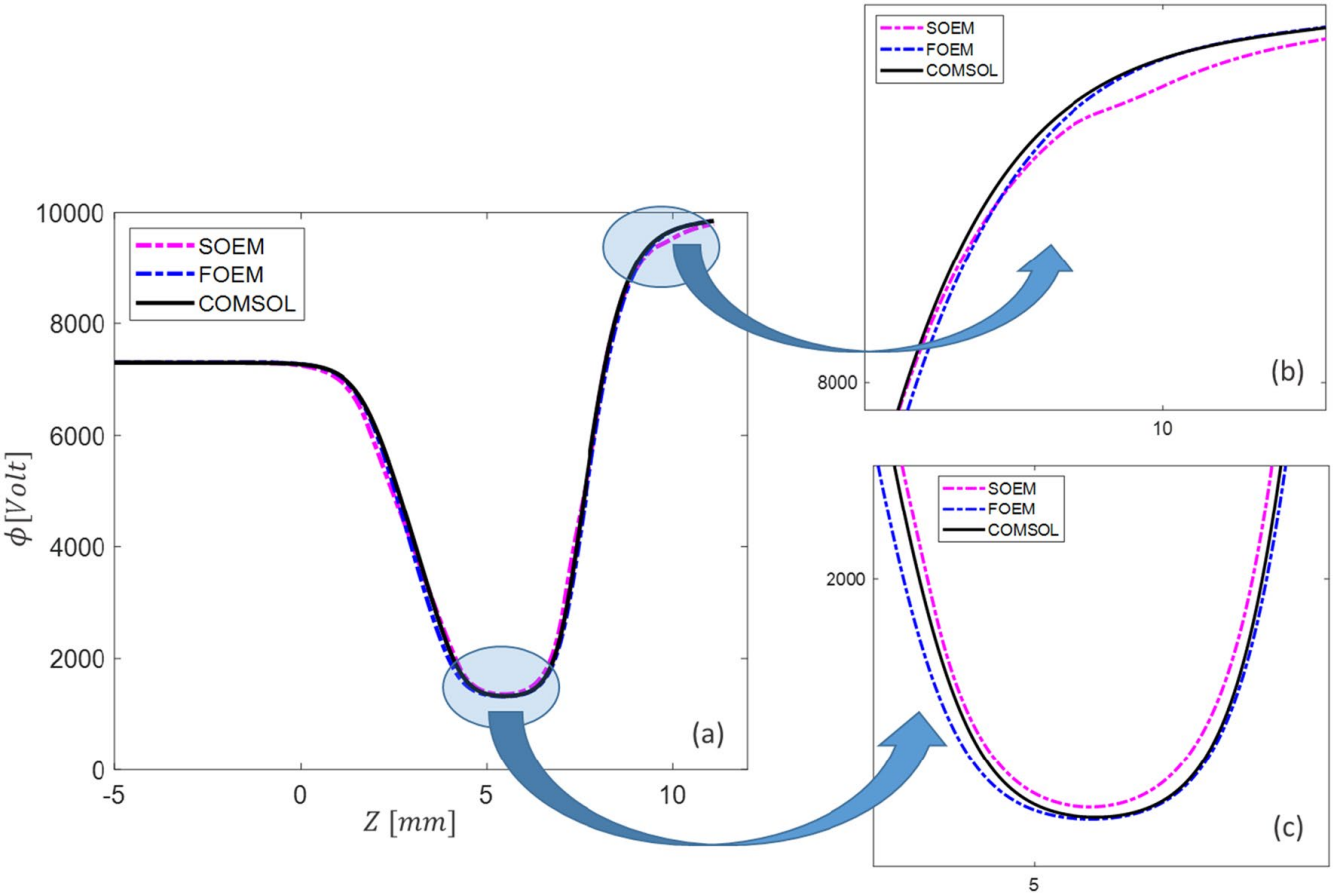
(**a**) The overlapped graphs of the axial potential for System 1 from Fig. 5, calculated with different methods of SOEM, FOEM and FEM. (**b**) and (**c**) the enlarged sections of the graphs.

**Figure 7:**
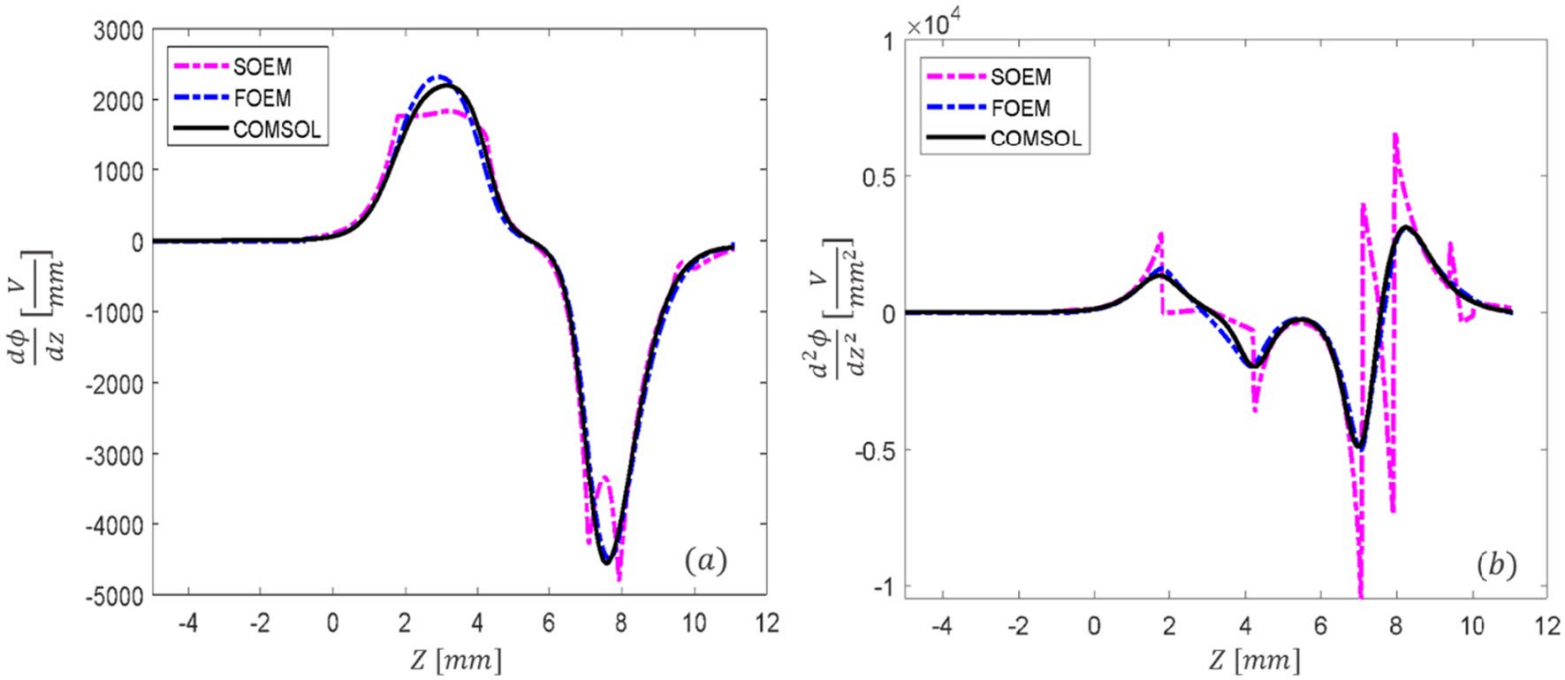
The overlapped graphs of the first (**a**) and second (**b**) derivative of the axial potential for System1 from Fig. 5, calculated with different methods of SOEM, FOEM and FEM.

To analyse the accuracy of calculation of the electric field and optical parameters using FOEM, six different typical electrostatic lens systems including 3, 4 and 5 electrodes, are taken as test systems (represented in 2D in Fig. 5). The axial potential calculation is performed by the two previously existing methods of SOEM and FEM (using COMSOL^13^) and by the newly-presented FOEM method. For the SOEM and FOEM calculation, we use MATLAB^34^. First, the axial potential and its derivatives are graphically compared. In the graphical comparison, only the graphs related to one system (system1 from Fig. 5) are depicted and presented here since all lens systems show almost similar trends. The overlaid graphs are presented in Figs. 6 and 7.

In electron optical lens systems, the aberration coefficients are the factors which determine the quality of the lens systems. The lower the values of these parameters, the less the aberrations exist and therefore the better the lens system^1–3^. In our case-study, the lens systems are only suffering from the spherical and chromatic aberrations. Hence, the chromatic and spherical aberration coefficients (denoted by $C_{c}$ and $C_{s}$) are the deterministic factors in the lens design and optimization here. These parameters are functions of the axial potential and its derivatives. The presentation of the formulation is given in Eqs. (48) and (49). More details on the formulations and their derivations are skipped in this paper and can be found in the references^7,12^.

48 $$C_{S} = \frac{1}{{16\phi_{0}^{\frac{1}{2}} }}\mathop \smallint \limits_{{z_{0} }}^{{z_{i} }} \phi^{\frac{1}{2}} \left( {\frac{5}{4}\left( {\frac{{\phi^{\prime\prime}}}{\phi }} \right)^{2} + \frac{5}{24}\left( {\frac{{\phi^{\prime}}}{\phi }} \right)^{4} r_{\alpha }^{4} + \frac{14}{3} \left( {\frac{{\phi^{\prime}}}{\phi }} \right)^{3} r_{\alpha }^{3} r_{\alpha }{\prime} - \frac{3}{2}\left( {\frac{{\phi^{\prime}}}{\phi }} \right)^{2} r_{\alpha }^{2} r_{\alpha }^{^{\prime}2} } \right)dz$$

49 $$C_{c} = \phi_{0}^{\frac{1}{2}} \mathop \smallint \limits_{{z_{0} }}^{{z_{i} }} \left( \frac{3}{8} \right) \frac{{\phi^{^{\prime}2} }}{{\phi^{\frac{5}{2}} }}r_{\alpha }^{2} dz$$

where $\phi_{0}$ is the potential at the object side, and $r_{\alpha } \left( z \right)$, refers to the principle imaging ray^1^ , travelling from the object side along the optical axis, with angle 1. The principle imaging ray $r_{\alpha } \left( z \right)$ is calculated by ray tracing.

**Figure 8:**
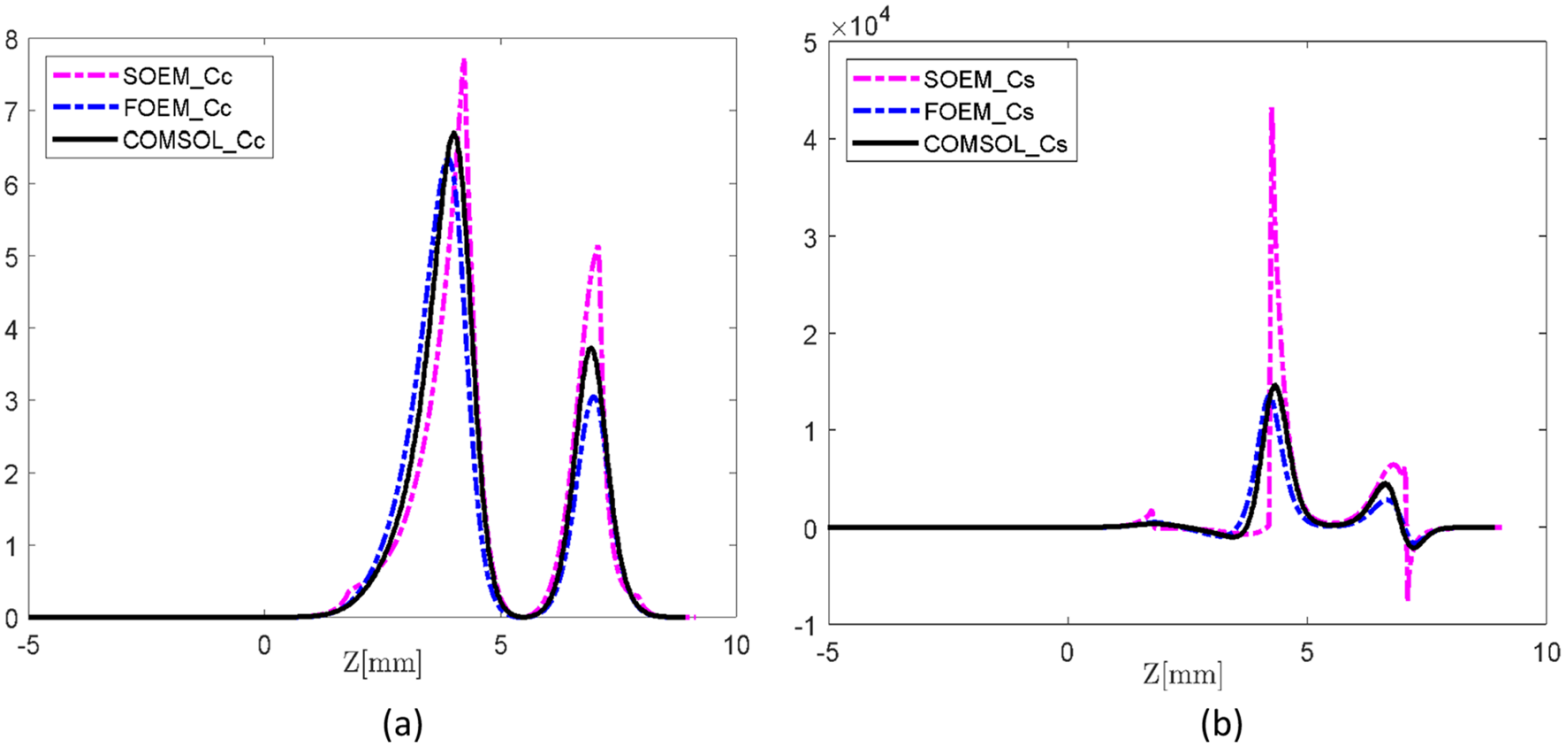
The overlapped graphs of chromatic (**a**) and spherical (**b**) aberration coefficients for System1 from Fig. 5, calculated with different methods of SOEM, FOEM and FEM. The unit of the y-axis is in millimeters.

The accuracy in the calculation of $C_{S}$ and $C_{c}$ is important in electron lens design. Since these parameters are functions of the potential and its derivatives, hence they can be used as an important qualitative and quantitative measure to check the accuracy of the axial potential calculations. Therefore, these optical parameters are also derived from the calculated axial potential and its derivatives by FOEM, SOEM and FEM and compared. The related graphs are presented in Fig. 8. The quantitative data related to these optical parameters are presented and compared in Tables 1 and 2.

**Table 1:** The data related to the optical parameters such as chromatic and spherical aberrations (*C*_*c*_ and *C*_*s*_) and the image position (*X*_*c*_), calculated by FEM, FOEM and SOEM, for six different typical electrostatic lens systems (presented in Fig. 5).

Systems	System1	System2	System3	System4	System5	System6
*X*_*c*_ (mm)						
FEM	8.92	8.26	9.65	13.39	10.03	15.43
FOEM	8.87	8.29	9.61	13.12	9.97	15.39
SOEM	9.12	8.62	9.87	13.78	10.35	15.65
*C*_*c*_ (mm)						
FEM	18.71	15.26	23.73	26.13	1.82	0.98
FOEM	18.85	15.35	23.45	27.79	1.89	0.94
SOEM	19.45	16.80	24.10	18.78	2.03	0.77
*C*_*s*_ (mm)						
FEM	21.13	54.70	22.88	217.65	7.14	12.48
FOEM	21.98	56.85	21.82	206.65	7.47	11.02
SOEM	30.77	104.58	37.07	392.57	13.19	11.77

**Table 2:** The percentage of error in calculation of the optical parameter derived by FOEM and SOEM, for six different typical electrostatic lens systems (presented in Fig. 5) compared with the data calculated with the accurate method of FEM.

Systems	System1 (%)	System2 (%)	System3 (%)	System4 (%)	System5 (%)	System6 (%)	Error avg. (%)
Error in *X*_*c*_							
FOEM	0.56	0.36	0.41	2.02	0.60	0.26	0.70
SOEM	2.24	4.36	2.28	2.91	3.19	1.42	2.73
Error in *C*_*c*_							
FOEM	0.75	0.59	1.18	6.35	3.85	4.08	2.80
SOEM	3.95	10.09	1.56	28.13	11.53	21.43	12.79
Error in *C*_*s*_							
FOEM	4.02	3.93	4.63	5.05	4.62	11.70	5.66
SOEM	45.62	91.189	62.01	80.37	84.73	5.69	61.60

Figure 6a, compares graphically the axial potential calculated by the three different methods (SOEM, FOEM and FEM), and a reasonably good overlap in the axial potential graphs can be seen. However, when zooming in (Fig. 6b,c, slight deviations are recognized. These deviations become more recognizable in the graph of $\phi^{\prime}$ (Fig. 7a), and $\phi^{\prime\prime}$ (Fig. 7b). It is evident that the deviations are mainly between SOEM and FEM, while there is reasonably good overlap between FOEM and FEM.

In the $C_{s}$ and $C_{c}$ graphs, shown in Fig. 8, similar to the graphs of the axial potential derivatives, the graphs of FOEM are in much higher agreement with FEM than the ones of SOEM. This behaviour is more recognizable in the $C_{c}$ graphs. This is because in our case-study, $C_{s}$ is dependent on both the first and second derivative of the axial potential, while $C_{c}$ is only a function of the first derivative, and the fluctuations in the second derivatives (as seen in Fig. 7a,b) are larger.

In addition to the graphical comparisons, to also achieve quantitative data comparisons, the values of optical parameters such as chromatic and spherical aberrations ($C_{c}$ and $C_{s}$) as well as the image position ($X_{c}$) (another important factor in lens design)^1^, are calculated for the six lens systems (from Fig. 5) and presented in Tables 1 and 2. The presented data of $C_{s}$ and $C_{c}$ are related to the image side.

From Table 1, it can be seen at a glance that all optical parameters, when calculated by FOEM are much closer to the accurate values calculated by FEM, compared to those calculated by SOEM. The difference between FOEM and SOEM in being more compatible with FEM, can be especially realized in $C_{s}$ values.

To estimate the deviation of the calculated data with FOEM and SOEM for each system, with respect to the accurate data, the error is determined by taking COMSOL data (FEM) as the reference for accurate values. The errors are presented in Table 2 for each system. The last column in Table1 presents the average value of the error for 6 lens systems. This average is computed as a mathematical mean of the errors over the six different lens systems ($(\sum\nolimits_{i = 1}^{6} {\left( {error_{i} } \right)}$)/6)).

As it is seen at a glance from Table 2, the reported errors from calculation with FOEM for all systems are much smaller than the ones calculated with SOEM. It should be noted that it might be possible to occasionally get a result with a slightly larger calculation error with FOEM than with SOEM. This scenario can occur due to the determination of the total values of $C_{s}$ and $C_{c}$ through the integration of summations involving terms dependent on $\phi$, $\phi^{\prime}$ and $\phi^{\prime\prime}$. Fluctuations are evident in the graphs of these parameters, particularly seen in Fig. 8a and b for phi' and phi". When examining the overlap of graphs from FOEM and SOEM with those from FEM in these figures, larger deviations are noticeable in SOEM compared to FOEM. However, in the calculations of $C_{s}$ and $C_{c}$, owing to the complexities of the functions, the terms in the integral may occasionally compensate for each other. This compensation results in the total values of $C_{s}$ and $C_{c}$ calculated for SOEM occasionally getting closer to those calculated with FEM compared to FOEM with FEM, even though the absolute values at each point in the graphs of $\phi$, $\phi^{\prime}$ and $\phi^{\prime\prime}$ for FOEM and FEM exhibit closer values. Such instances, however, are infrequent. In general, as shown in Table 2, the calculated data by FOEM consistently demonstrates much smaller errors compared to SOEM.

As illustrated in Table 2, FOEM values provide reasonably accurate results for $X_{c}$ (with the average value of error 0.7%), with respect to the accurate values of FEM. Also, noticeable is that, FOEM resulted in much smaller error percentages (approx. 4 times smaller) than SOEM with respect to the accurate values. A similar trend of having higher accuracy for FOEM than SOEM is seen in the values of $C_{c}$. The error percentage, on average, for $C_{c}$ calculation by FOEM is around 5 times smaller than by SOEM (i.e. 2.8% for FOEM versus 12.79%). Having much smaller error percentage (~ 4–5 times) for $C_{s}$ calculation in FOEM compared to SOEM (5.6% for FOEM versus 61.6%) keeps the same trend of higher accuracy of FOEM. However, the absolute values of the error percentages in $C_{s}$ are much higher than in the cases of $C_{c}$ and $X_{c}$ calculation. which is due to their different functionality to the potential and its derivatives. The computation time needed to evaluate the optical parameters for each lens system, using FEM, FOEM and SOEM are ~ 60 s, 0.5 s, and 0.4 s, respectively.

In summary, the quantitative data comparison, similar to the graphical analysis, demonstrates that FOEM performs the optical parameters calculation in a very short time with reasonably high accuracy, showing deviations ranging from around 1% to a maximum of 6% compared to FEM. Notably, FOEM exhibits significantly higher accuracy in both field calculation and optical parameter calculations compared to SOEM. This is particularly evident for optical parameters dependent on the second derivative of the potential, such as $C_{s}$.

## Optimization of electrostatic electron lens systems based on FOEM and a comparison with SOEM- and FEM-based optimization

As discussed earlier, the primary goal of developing a fast field calculation method for electrostatic lens systems was to incorporate it into an automated optimization routine for lens system optimization. With the identification of the proposed fast field calculation method (FOEM), the next step is to implement it in an optimization routine (here Genetic Algorithm) to assess its efficiency in producing optimized lens systems with accurately calculated optical parameters, all within a reasonably short computational runtime. This objective will be addressed in this section.

In this section therefore first a brief introduction on Genetic Algorithm (GA) in the context of electrostatic electron lens system optimization is provided in “Introduction to electrostatic electron lens optimization by FOEM + GA” section. Then a section is introduced (in “The calculation of optical parameters in a single run of GA Optimization based on FOEM, versus SOEM and FEM”), to conduct an optimization that calculates the potential using FOEM (referred to as 'FOEM + GA') and verify the accuracy of the calculated optical parameters associated with the resulting lens systems within a single run of GA. The aim of this section is to analyse the accuracy and trends in optical parameter calculation using FOEM, SOEM, and FEM in a single run of GA Optimization.

The problem in comparing the outcomes of the three methods is that they all may yield small values for the optimization parameter, but the calculation of the parameters by SOEM and FOEM may be inaccurate and when the parameters of the resulting optimized designs are calculated by FEM, they may be much higher. The solution to that problem is of course to calculate the optical parameters of the resulting designs by FEM.

There is one more check we need to do in order to have a fair comparison between FOEM and SOEM. In a previous study^7,12^, we followed a SOEM optimization by a FEM optimization that was fed by the results of the SOEM optimization. This turned out to be very efficient as compared to a strict FEM optimization. Now the question is if a strict FOEM optimization, or a FOEM optimization with subsequent FEM optimization can compete with that earlier approach. A detailed exploration of the aforementioned subjects is presented in “The efficiency of GA optimization based on FOEM, FEM and SOEM” section.

### Introduction to electrostatic electron lens optimization by FOEM + GA

The Genetic Algorithm (GA), a member of the evolutionary algorithms, emulates natural evolution, drawing inspiration from Darwin’s theory. The process commences with the creation of a randomly generated initial population denoted as $P_{1} \left( {x_{1} , \ldots , x_{n} } \right)$ where '$x_{i}$' signifies the chromosomes in natural evolution and, in the context of electrostatic electron lens system optimization, represents the electrostatic electron lens systems. Consequently, $P_{1}$ constitutes an initial set of randomly generated electrostatic electron lens systems based on the variables of the lens system. In the context of genetic algorithms (GA), the user has the option to provide some or all of the initial elements of the first population instead of implementing solely the randomly generated ones.

In natural evolution, over time, the initial population gradually evolves towards members that are better adapted to their environmental conditions. Likewise, in the GA, the initial population progresses through successive "generations" towards a new set of systems $\left[ {P_{i + 1} \left( {x_{1} , \ldots , x_{n} } \right)} \right]$ that better satisfy the problem conditions. The conditions identified for optimization are specified by a designated “objective function”. The term “generation” in the GA corresponds to the number of iterations the algorithm undergoes before termination. In this study, this parameter serves as the stopping criterion for the GA. The GA is determined by also other tuning operators such as "crossover," and "mutation."

### The calculation of optical parameters in a single run of GA optimization based on FOEM, versus SOEM and FEM

To undertake this analysis, a full optimization is performed utilizing the Genetic Algorithm to optimize a four-electrode electrostatic lens system, with optical parameters calculated using the FOEM technique. Based on insights from our recently published work on tuning GA parameters for electrostatic lens system optimization^21^, also considering the fact that the primary goal of this investigation is not to obtain the best-optimized system but rather to derive results for comparing different field calculation approaches implemented within GA, the chosen GA tuning parameters are as follows: population = 50, generation = 100, Crossover :”Heuristic” and Mutation: “Gaussian” techniques, with a crossover fraction of 0.6 (indicating the proportion of the population through which the next generation is created by the crossover function) and the mutation rate of 0.4.

All the geometries of the lenses, such as thicknesses ($T_{j}$), radii ($R_{j}$) and gaps between electrodes ($G_{j}$), as well as the voltages at each electrode ($V_{{EL_{j} }}$) are free parameters of the optimization. These parameters can range in the interval of $1 \,{\text{mm}} < T_{j} \left( {j = 1, \ldots , 4} \right) < 3 \,{\text{mm}}$, $0.3 \,{\text{mm}} < R_{j} \left( {j = 1, \ldots , 4} \right) < 2\,{\text{mm}}$, $1\,{\text{mm}} < G_{j} \left( {j = 1, 2} \right) < 3\,{\text{mm}}$ and $700\,{\text{V}} < V_{{EL_{j} }} < 10\,{\text{kV}}\; \left( {j = 2, 3} \right)$. The voltage of the first electrode is 7000 V. In total, there are 12 free lens parameters in the optimization. The voltage at the image plane is kept fixed at 1000 V. The total length of the lens system is kept constant (here 15 mm). A schematic drawing of such a lens system can be seen in Fig. 5 (System4). The origin of the x-coordinate is at the left side of the first electrode’ s surface. The primary beam comes from far behind the origin (− 100 mm), to focus at the image plane. During the optimization the constraints are to have a fixed image position (at $X_{C} = 15 \;{\text{mm}}$, with a tolerance of ± 0.05), and to have a maximum electric field < 15 kV/mm (to avoid the voltage breakdown between sequential electrodes). The optimization objective is to obtain systems with the lowest value of the spherical and chromatic aberrations. The objective function is therefore a function of $C_{S}$ and $C_{C}$. How to define the objective function based on $C_{S}$ and $C_{C}$ can be different and depends on the application. Here, a summation of $C_{C}$ and $C_{S}$, with weights of 5 and 1, respectively, is taken. The objective function can be mathematically written as:

50 $$Objective\;function = 5 \cdot C_{C} + C_{S}$$

The abovementioned choice of the geometry, objective function and constraints, resembles an earlier reported probe-forming electrostatic objective lens system^5^. However, since the aim is to compare the results of “FOEM + FEM + GA” with “SOEM + FEM + GA”, this specific selection does not influence the results of the analysis and can also be altered with other logical choices.

**Figure 9:**
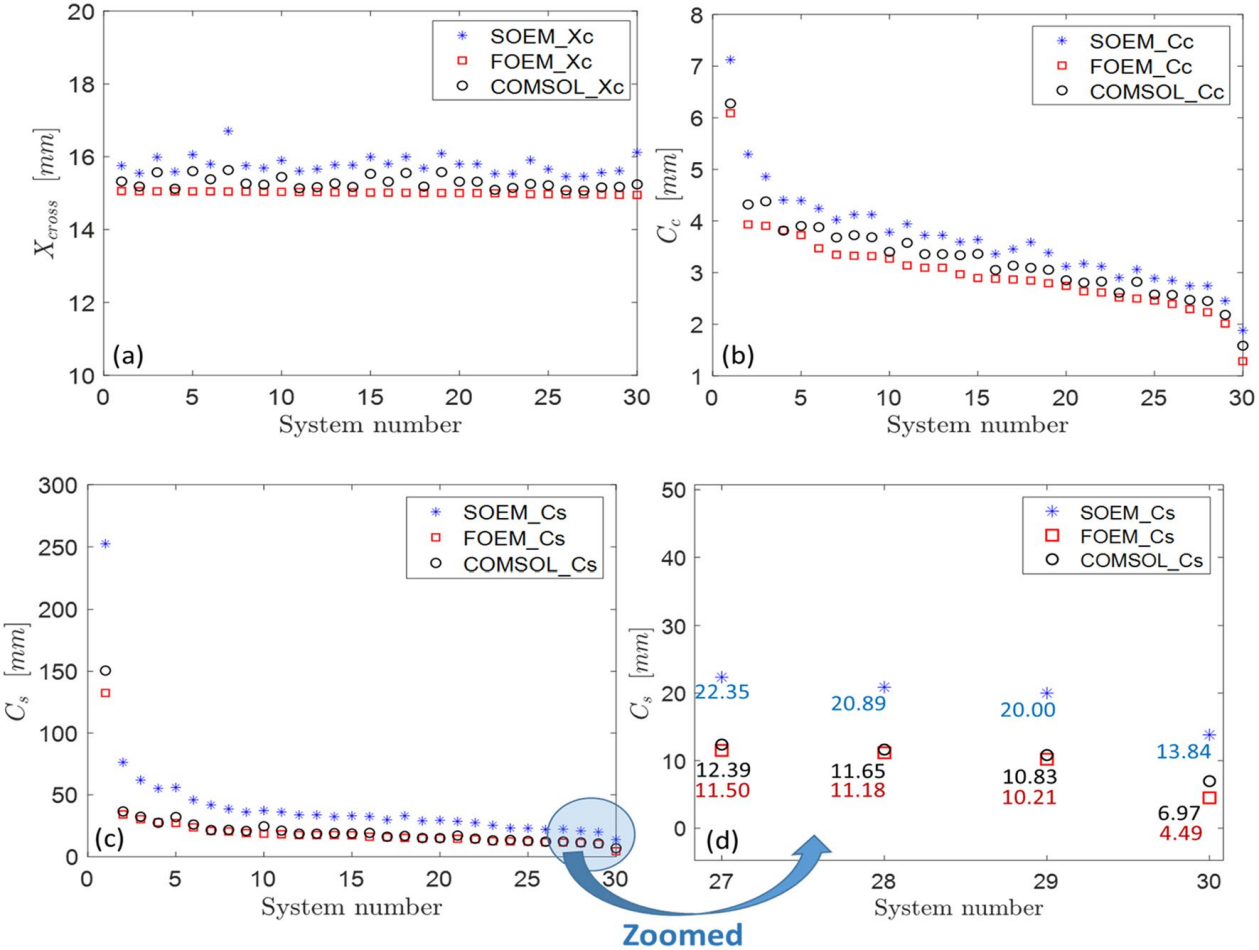
(**a**) Comparison of crossover point ($X_{C}$), (**b**) chromatic aberation cofficient ($C_{C}$), (**c**,**d**) and spherical aberration coefficient ($C_{S}$), for the best 30 systems obtained using SOEM, FOEM and COMSOL. The 30 systems are the best non-identical optimized systems derived from an optimization using GA with data calculation by FOEM.

Note: Our objective in this section is not to run a GA to assess its performance or to optimize and evaluate its effectiveness. Instead, we intend to generate several lens systems, surpassing the six systems listed in Table 1, each with distinct geometries. The primary goal is to reevaluate the accuracy of calculations for $C_{S}$, $C_{C}$, and $X_{C}$ by FOEM compared to FEM and SOEM across a broader range of lens systems. Additionally, we aim to investigate how the trend of decreasing $C_{S}$ and $C_{C}$, observed in FOEM calculations, translates when the same lens systems are computed using FEM. Specifically, we want to understand whether $C_{S}$ and $C_{C}$ maintain a similar trend of decrement when they are calculated by FOEM (compared to when they are calculated by FEM). This evaluation serves two purposes: firstly, to confirm the accuracy of $C_{S}$, $C_{C}$, and $X_{C}$ calculations for various systems, not just those in Table 1, and secondly, to find out whether lens systems with identical $X_{C}$ values with different $C_{S}$ and $C_{C}$ values when calculated by FOEM, would show a similar trend of decrement of optical parameters when using FEM. If that can be established, it suggests that FOEM is a potentially valuable method to be implemented in optimization routines for optical parameter calculations, and it would guide us towards systems with a decrement in their $C_{S}$ and $C_{C}$, even if there are some deviations in the accurately calculated values of $C_{S}$ and $C_{C}$. Simultaneously, a similar comparison is also performed for SOEM, allowing us to compare the results when they were calculated by FOEM compared to SOEM. To conduct this analysis, we could have manually found 30 diverse lens systems, each with $X_{C}$ at 15 mm. However, the task to manually create these test lens systems is challenging. To address this, instead, we utilized a GA run, incorporating a constraint of $X_{C} = 15\;{\text{mm}}$. Subsequently, we selected 30 lens systems that met the constraint and presented varying geometries. This automated approach allowed us to efficiently find 30 systems with decreasing $C_{S}$ and $C_{C}$ values, facilitating our evaluation. We didn't include all 5000 systems from their respective generations in the graph since they were too crowded. Instead, we manually selected the 30 best non-identical systems—those with the lowest objective function values and different designs—for this comparison. The reason for not selecting the real 30 best systems out of the GA run was to avoid having almost 30 identical systems that GA converged to as solutions in the last generations. This would have hindered a proper observation of the trend of decrement. Additionally, not having 30 almost very different lens systems would not provide a suitable basis for testing optical parameter accuracy calculations. Therefore, we manually chose 30 systems to represent the overall trend of decrement in their objective functions. These systems were sorted in the plot and identified by their sorted system numbers (System 1–30) in Fig. 8.

For the optimization, a GA is used with the generation and population of 100 and 50, respectively. Therefore, a total of 5000 systems are being evaluated in this optimization. From the results of this optimization, the 30 best non-identical systems (the system with the lowest objective function values with different designs) are taken for the comparison analysis. The related data are presented in Fig. 9.

The data of $X_{C}$, $C_{C}$ and $C_{S}$, for these 30 systems are given as squares (in red) in Fig. 9a–c, respectively. The optical parameters of $X_{C}$, $C_{C}$ and $C_{S}$ for these 30 systems are again calculated using SOEM and FEM (by COMSOL). The data calculated by SOEM are presented with stars (in blue), and by FEM are shown with circles (in black) in the same Fig. 9a–d). As it is clear from Fig. 9a, the data for $X_{C}$ are reasonably accurate both for FOEM and SOEM compared to FEM, but with higher accuracy for FOEM than for SOEM. It is noticeable from Fig. 9b–d, that the data of $C_{C}$ and $C_{S}$ for FEM and SOEM follow the same decreasing trend as FOEM, however, with much less deviation from the accurate data of FEM for FOEM data compared to those of SOEM.

The first conclusion is that both SOEM and FOEM can be used in an initial optimization to start from no initial data and reach reasonably optimized systems. However, it is important to note that the inaccuracy in the data obtained with SOEM is large, specifically in $C_{S}$ (see Fig. 9c and zoomed-in in Fig. 9d).

### The efficiency of GA optimization based on FOEM, FEM and SOEM

**Figure 10:**
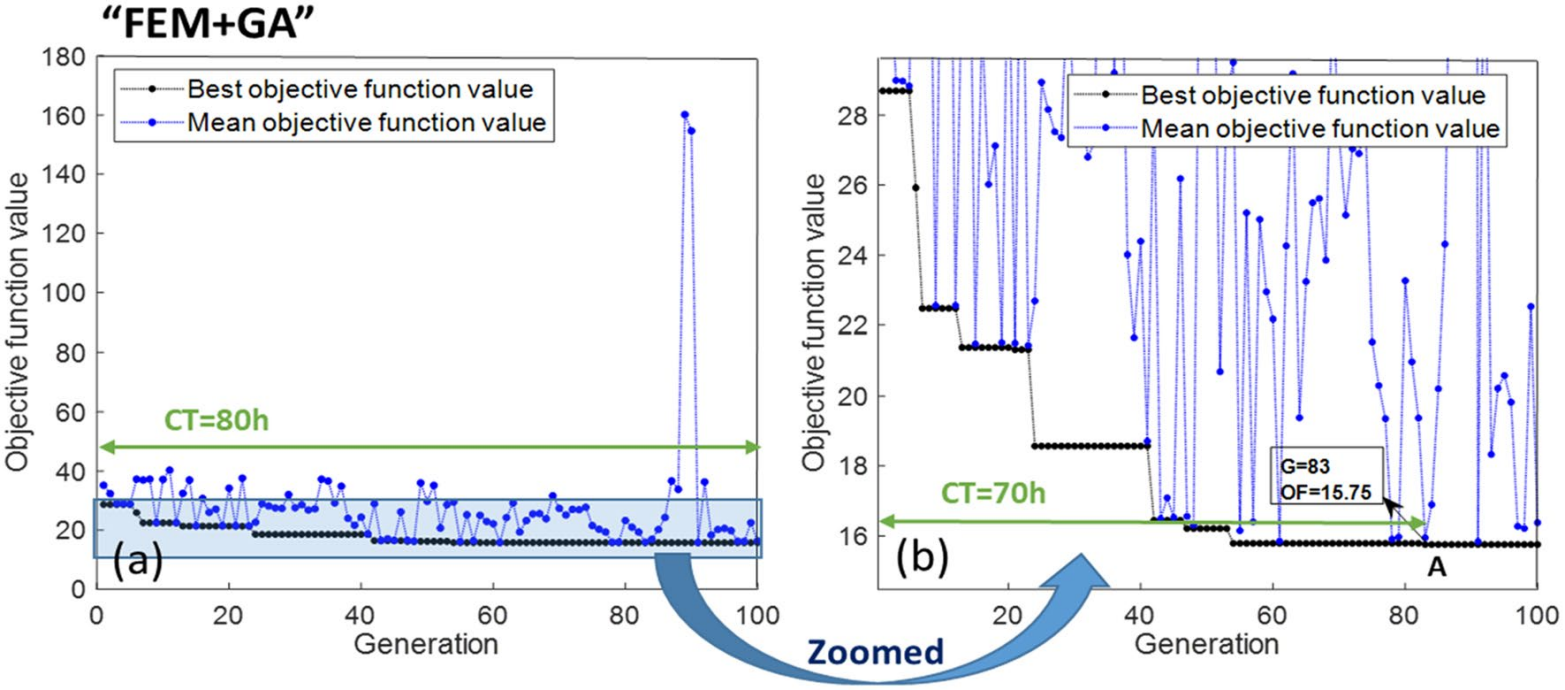
(**a**) Optimization of a 4-electrode lens system using GA, while the optical parameters are calculated by FEM (“FEM + GA”). The graph represents the objective function (OF) values (Y-axis) versus generation (X-axis). The black/blue dots show the best/mean objective function values at each generation. Figure (**b**) shows the zoomed-in data of figure a. System A is the best system found in this optimization with OF = 15.75, occurring at generation 83. ’CT’ stands for the computation time needed to run the optimization till the generation indicated by the green arrow.

**Figure 11:**
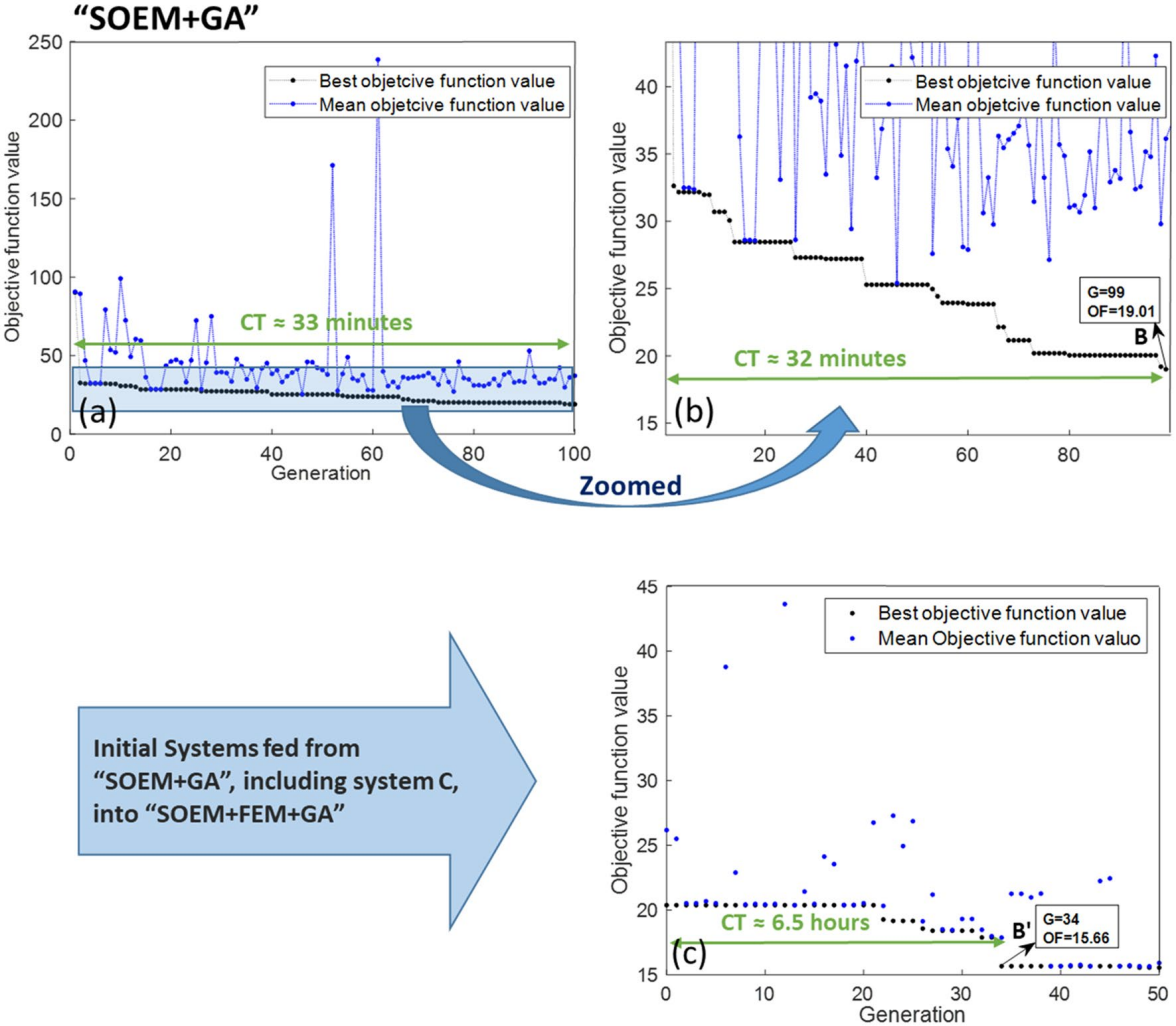
(**a**) The results from an optimization of a 4-electrostatic lens system using GA, while the optical parameters are calculated by SOEM (“SOEM + GA”). The graph represents the objective function (OF) values (Y-axis) versus generation (X-axis). The black/blue dots show the best/mean objective function values at each generation. Figure (**b**) shows the zoomed-in data of figure (**a**). System B is the best system found in this optimization. ’CT’ stands for the computation time needed to run the optimization until the generation indicated by the green arrow. Figure (**c**) presents the result of an optimization using GA, called “SOEM + FEM + GA”, with the initial data fed from a previous optimization of “SOEM + GA”. A lens system, depicted by B’, is an optimized system resulting from “SOEM + FEM + GA” with OF = 15.66, occurring at generation 34.

**Figure 12:**
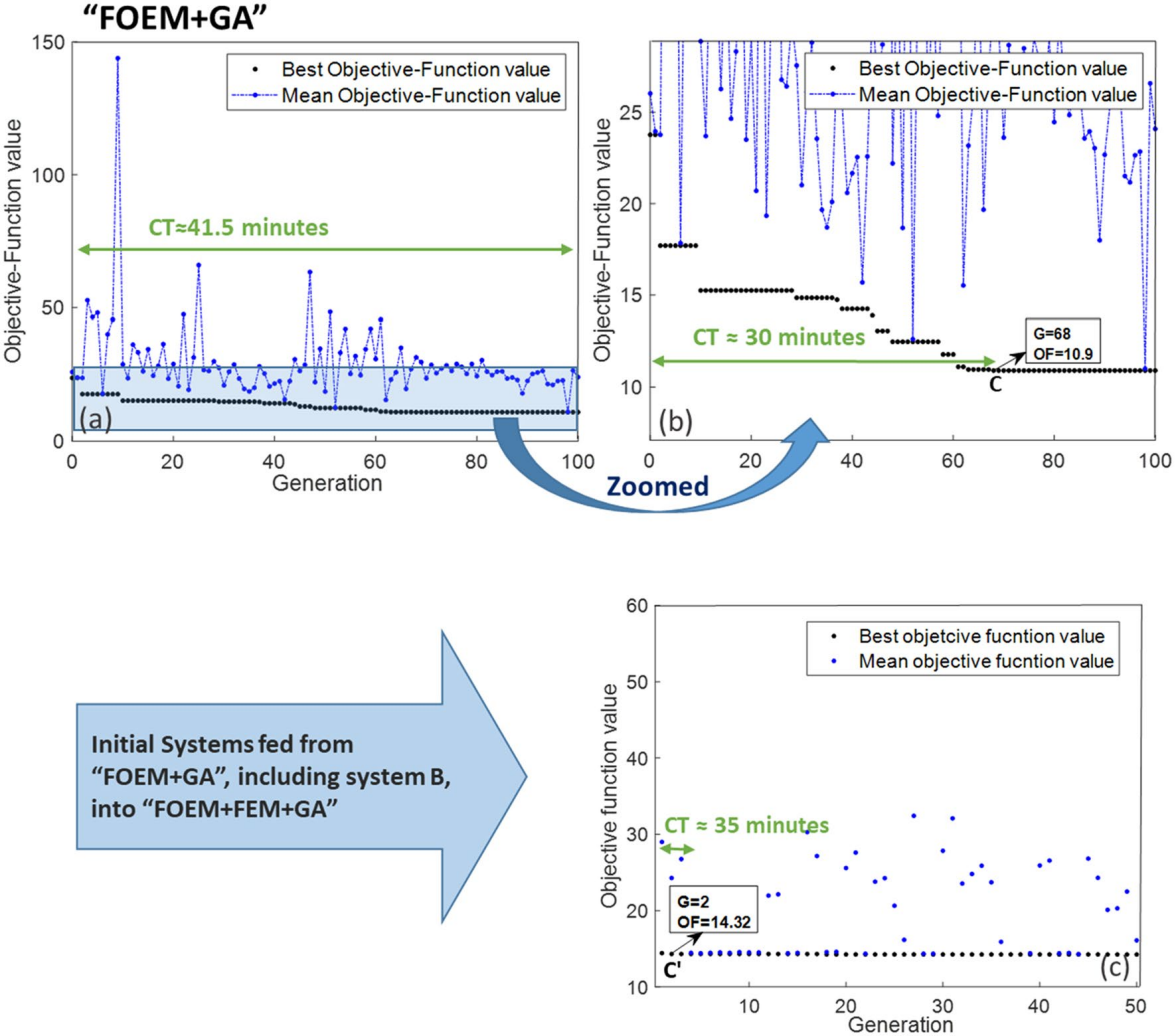
(**a**) The results from an optimization of a 4-electrostatic lens system using GA, where the optical parameters are calculated by FOEM (“FOEM + GA”). The graph represents the objective function (OF) values (Y-axis) versus generation (X-axis). The black/blue dots show the best/mean objective function values at each generation. Figure (**b**) shows the zoomed-in data of figure (**a**). System B is the best system found in this optimization. ’CT’ stands for the computation time needed to run the optimization until the generation indicated by the green arrow. Figure (**c**) presents the result of an optimization using GA, called “FOEM + FEM + GA”, with the initial data fed from a previous optimization of “FOEM + GA”. A lens system depicted by C’, is an optimized system resulting from “FOEM + FEM + GA” with OF = 14.32, occurring at generation 2.

In this subsection, the objective is to compare the outcomes of FOEM + GA, FEM + GA, and SOEM + GA. To achieve this, different optimizations, as detailed below, will be executed and subsequently compared: FEM + GA optimization starting with random parameters (very time consuming), then FEM + GA starting with a feed from SOEM and finally FEM + GA starting with a feed from FOEM. The generation (denoted by ‘G’) and population (denoted by ‘P’) in GA are taken as 100 and 50, respectively. The results of these three optimizations are presented in Fig. 10a, 11a and 12a. Figures 10b, 11b and 12b depict a zoomed-in view. As previously discussed, the results from FEM are accurate. Therefore, the results taken from “FEM + GA” do not need to be fine-tuned. In order to get accurate and optimized out-put results, the 10 best optimized systems are taken and fed into another optimization by GA as its initial population, but this time the objective function is calculated using FEM. The results of these optimizations which are called “SOEM + FEM + GA” and “FOEM + FEM + GA”, are presented in Figs. 11c and 12c, respectively. The quantitative data related to these optimizations are represented and summarised in Table 3.

**Table 3:**
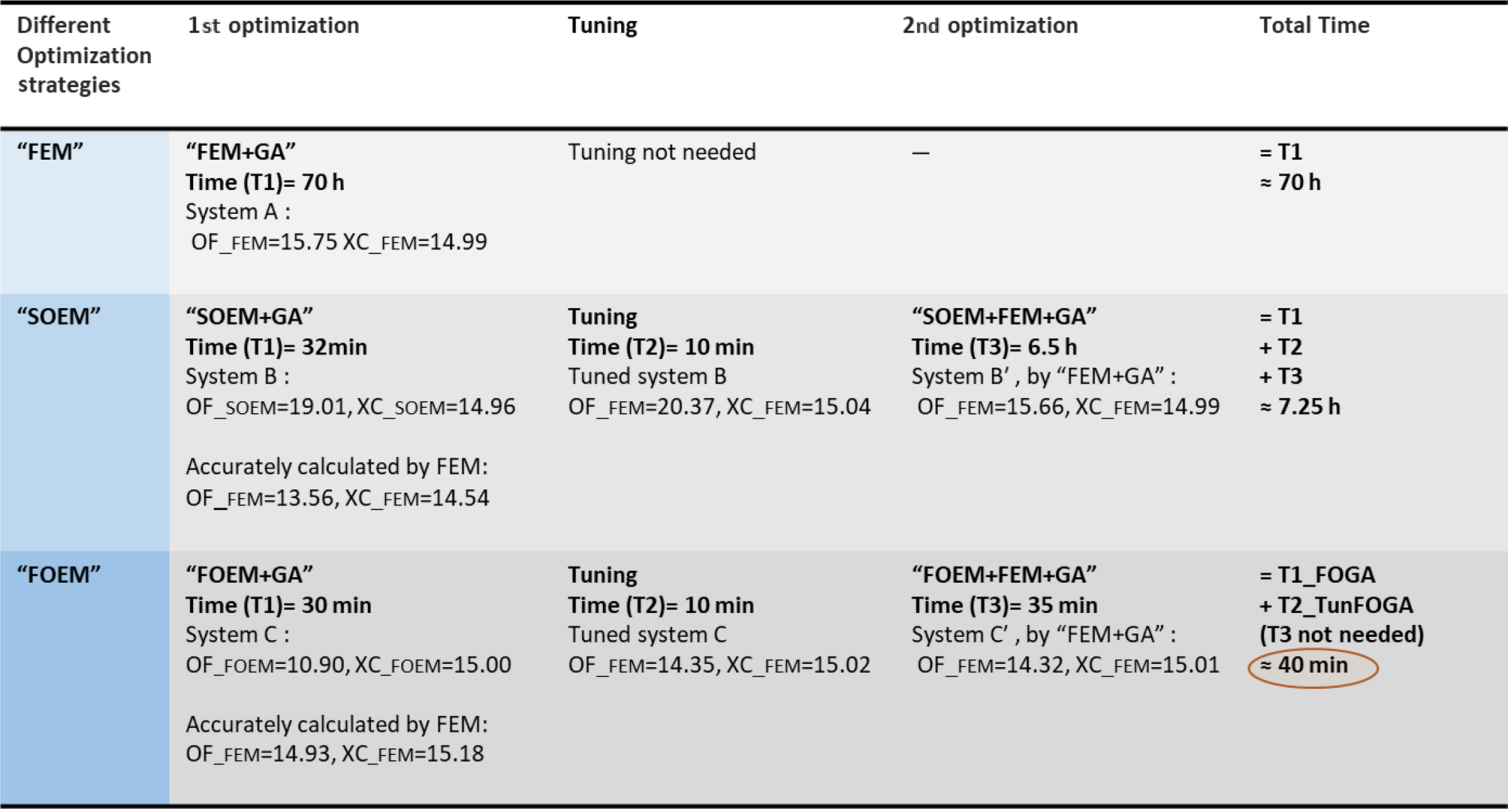
The data related to the comparison between three optimization strategies of “FEM + GA”, “FOEM + FEM + GA” and “SOEM + FEM + GA”.

As is seen from Fig. 10a, to run the full optimization for “FEM + GA”, the computation time (denoted by CT in the Fig. 10, 11 and 12), is 80 h. However, the best system, with the objective function of 15.75 (shown by ‘A’ in Fig. 10b), is reached at generation G = 83. Hence, the computation time needed to reach the best system in this case is around 70 h. After this time, the optimization could not be improved any further within its 100 generations. Therefore the CT needed for “FEM + GA” can be reported as 70 h and the best system has OF = 15.75 (represented in Table 3 as well).

Figure 11, shows the results of “SOEM + FEM + GA”. The first plot, 9a, presents the improvement of the objective function versus generation during the optimization. The total computation time to run this optimization is CT ~ 33 min. The zoomed-in data is shown in Fig. 11b where it is seen that the optimization has improvements almost until the last generation. The best system (system B in Fig. 11b) is found at generation of G = 99, with an objective function of OF = 19.01. The computation time to find this system is ~ 32 min. The related data for this system is given in Table 3. As is evident from Table 3, the image position of this system when derived from “SOEM + GA” is reported to be $X_{C} \_SOEM$ = 14.96 mm. However, when the optical parameters of this system are calculated accurately by FEM, $X_{C} \_FEM$ = 14.54 mm and $OF\_FEM$ = 13.56 is achieved.

Here OF_FEM seems to have a small value of 13.56. However, it should be noted that the accurate Xc when calculated by FEM turned out to be $X_{C} \_FEM$ = 14.54 and not at the expected required value of $X_{C }$ = 15 mm. It means, this system is not at the required fixed-image position and might have a larger OF value, when it is tuned to have its $X_{C }$ at 15 mm.

This system, together with nine other optimized systems, are fed into another optimization of “FEM + GA” (with P = 20, G = 50), as their initial population. The optimized system (B’ in Fig. 11c), is found at generation of G = 34. The optimization did not show recognizable improvement afterwards. Therefore G = 34 is taken to be the generation at which the optimization reached the best system. The computation time to reach this system is ~6.5 h. Figure 11c shows that the best system found by SOEM actually had an OF = 21 when properly focused instead of the values OF = 19.01 or 13.56 as discussed above. The FEM optimization could improve the design to OF = 15.66.

To complete the comparison, an optimization is performed using FOEM. The results of this optimization are presented in Fig. 12a. It is evident that the computation time for a full optimization is around 41.5 min. The best system (system C with OF = 10.9) here is found at G = 68 after around 30 min. No further improvement is recognized after that.

As seen from Table 3, the image position and objective function values of system C derived by “FOEM + GA” are $X_{C} \_FOEM$ = 15.00 mm, and $OF\_FOEM$ = 10.9. These parameters when they are calculated accurately by FEM are reported to be $X_{C} \_FEM$ = 14.93 mm and $OF\_FEM$ = 15.18 (see Table 3). To achieve the accurate and optimized out-put system, this optimized system (system C in Fig. 12b), together with nine other best systems are taken and fed into another optimization of “FEM + GA”, with P = 20, G = 50 as the initial population. The result of this optimization is presented in Fig. 12c. As is evident from Fig. 12c, the optimization already reached the optimized system (system C’ in Fig. 12c) after only the first generation (at G = 2, OF = 14.32). After the second generation the optimization improvements are almost negligible. This means that the initial systems which had been fed were close to the accurate solutions calculated by FEM.

The reason why the ”SOEM + FEM + GA” optimization takes a longer time than “FOEM + FEM + GA” to find the optimized system is that the objective function and image position of the optimized system found by this optimization (system B) deviate more from the corresponding accurately calculated values by FEM (see Table 3) than those achieved by “FOEM + GA” (system C).

In summary, based on what has been discussed above and reported in Table 3, it is seen that the total computation time needed to run a fully-automated optimization, for “FEM + GA”, “SOEM + FEM + GA” and “FOEM + FEM + GA”, is 70 h, 7 h and 1.25 h (i.e. 35 min + 40 min), respectively. The optimized systems found by these three methods, which satisfied the constraints, have objective function values of 15.75, 15.66 and 14.32, respectively. Although “SOEM + FEM + GA” could reach an optimized system with low objective function within a reasonably short time (~ 7 h), “FOEM + FEM + GA” could reach it ~10 times faster and it is even ~70 times faster than the optimization based on “FEM + GA”. From these results, it is clear that “FOEM + FEM + GA” could reach a lower value of the objective function and in an enormously shorter computation time than the other two optimization strategies.

However, what is noticeable here is that, as it is seen from the last row of the Table 3, the optimized system found by only “FOEM + GA” got close enough to the accurate optimized system, which after a very short time of tuning (10 min) could achieve an optimized system which could not be further improved by a full optimization of FEM + GA (after 35 min of “FOEM + FEM + GA”, the optimized system has the optical parameters of: OF_FEM = 14.32 with XC_FEM = 15.01 mm, resulting from improving on a previously optimized system of : OF_FEM = 14.35, XC_FEM = 15.02 mm).

Hence, it is evident that FOEM results are close enough to the accurate results such that this method has the possibility to be even used separately in an optimization run without the need of another full run of FEM, while SOEM needed that. This method therefore can have high impact on the process of automated design and optimization of electrostatic lens systems.

**Figure 13:**
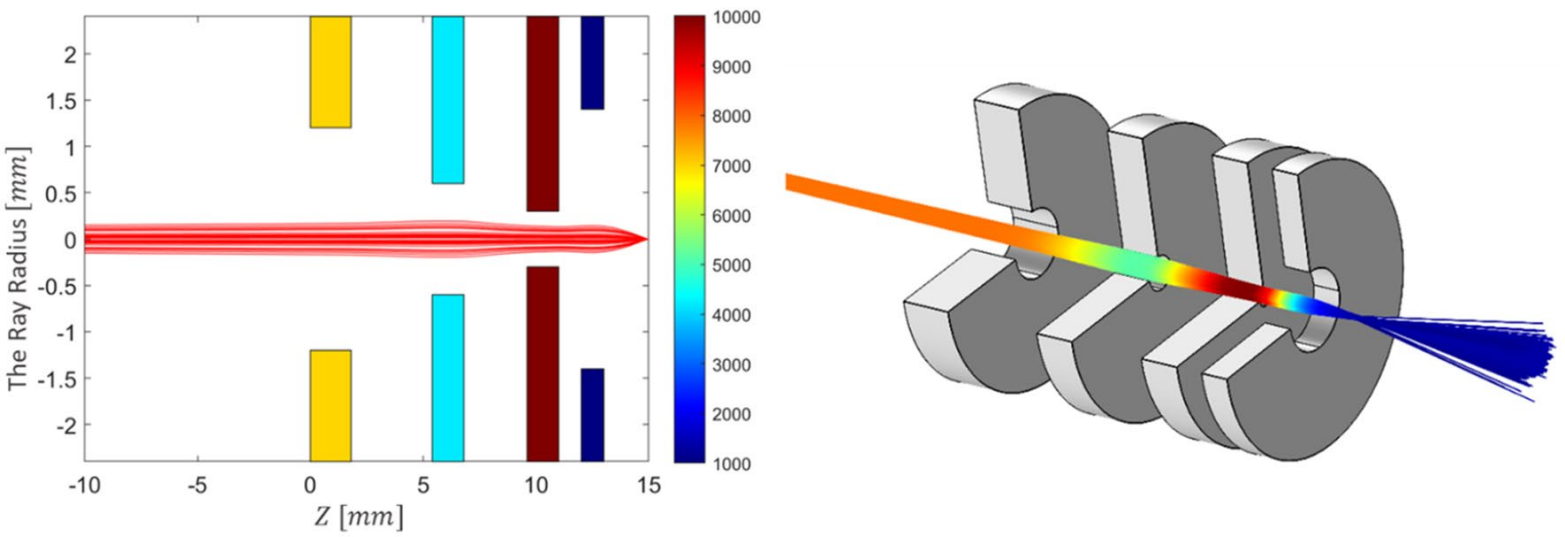
The optimized electrostatic lens system (system C’ in Fig. 12c) including 4 electrodes, optimized by “FOEM + GA”, in 2D (**a**) and 3D (**b**).

To have a visualisation of the electrostatic lens system optimized by “FOEM + GA”, system C’ is presented in Fig. 13 (2D in a, 3D in b).

## Conclusion

In this work, a novel fast field calculation method, with reasonably high accuracy, for cylindrical rotationally-symmetrical electrostatic lens systems is presented. It is performed by solving the Laplace equation while keeping the terms up to its fourth order derivatives and utilizing quintic splines. This method is therefore called Fourth Order Electrode Method (FOEM) by the authors. First, fundamental equations of the quintic spline are derived for a system with unequal distances between the points, which is new as far as we know. Using the Laplace equation and by means of discretizing the axis-of-symmetry and approximation of the axial potential in each section with a quintic spline, a mathematical equation for calculating the potential along the axis of symmetry is derived. Accuracy and computation time of the proposed method in calculation of the axial potential, its first and second derivatives and related optical parameters, are compared with other field calculation methods, such as the Finite Element Method (FEM) and Second Order Electrode Method (SOEM). As expected, especially the second derivative of the axial potential is calculated much more accurate in FOEM than in SOEM, which is especially important for the calculation of the spherical aberration of a lens system.

The proposed method is then used to optimize an electrostatic lens system by implementing Genetic Algorithm (GA) optimization and the computation time of optimization and effectiveness of evaluated optical parameters are compared with optimization performed by “FEM” as well as “SOEM + FEM”. It is concluded that using “FOEM”, optical parameters calculated by FOEM achieve reasonably high accuracy compared with values obtained by FEM and the optimization based on FOEM can be performed significantly faster (70 times) than by FEM. When the final design found by FOEM optimization is recalculated by FEM, the resulting lens parameters are very close to the results from the much slower FEM optimization.

When the results from the FOEM optimization are fed into the GA optimization with FEM field calculation, it is found that the results only improves marginally. For other systems than our example, this may be different, but based on the experience in the analysed example it is expected that such a “fine tuning” step can be quite short.

This new method might be of interest to electron optics designers when a fast calculation of the axial field in electrostatic lens systems is needed. The most obvious application is the fully-automated design and optimization of multi-electrode lens systems, with many free parameters and multi-objective functions.

### Supplementary Information

Supplementary Information.

## Data Availability

All data generated or analysed during this study are included in this published article and its supplementary information file.
