# Supplementary material for: A new approach for fast field calculation in electrostatic electron lens design and optimization
Source: Sci Rep. 2024 Feb 28;14:4859. doi: 10.1038/s41598-024-55518-3 (PMC11319647; doi:10.1038/s41598-024-55518-3)
Supplement: Supplementary file 1 — Supplementary Information. [file 41598_2024_55518_MOESM1_ESM.docx]

**Appendix**

Matrix Representation of Eq. 38 :

$\left[ A \right]_{(N-2)\times N}{[Z]}_{N\times1}=\left[ B \right]_{(N-2)\times N}{[\mu]}_{N\times1}$

$$\left( \begin{matrix} \begin{matrix} a_{11} & a_{12} & a_{13} \\ & a_{22} & a_{21} \\ & & a_{33} \end{matrix} & \begin{matrix} & & \\ a_{23} & & \\ a_{34} & a_{35} & \end{matrix} & \begin{matrix} & & \\ & & \\ & & \end{matrix} \\ \begin{matrix} & & \\ & & \\ & & \end{matrix} & \begin{matrix} & & \\ & & \\ & & \end{matrix} & \begin{matrix} & & \\ & & \\ & & \end{matrix} \\ \begin{matrix} & & \\ & & \\ & & \end{matrix} & \begin{matrix} & a_{\left( N-4 \right)\left( N-4 \right)} & a_{\left( N-4 \right)\left( N-3 \right)} \\ & & a_{\left( N-3 \right)\left( N-3 \right)} \\ & & \end{matrix} & \begin{matrix} a_{\left( N-4 \right)\left( N-2 \right)} & & \\ a_{\left( N-3 \right)\left( N-2 \right)} & a_{\left( N-3 \right)\left( N-1 \right)} & \\ a_{\left( N-2 \right)\left( N-2 \right)} & a_{\left( N-2 \right)\left( N-1 \right)} & a_{\left( N-2 \right)\left( N \right)} \end{matrix} \end{matrix} \right)_{\left( N-2 \right)\times N}\left( \begin{matrix} \begin{matrix} Z_{1} \\ Z_{2} \\ Z_{3} \end{matrix} \\ \begin{matrix} \vdots\\ \vdots\\ \vdots\end{matrix} \\ \begin{matrix} Z_{N-2} \\ Z_{N-1} \\ Z_{N} \end{matrix} \end{matrix} \right)=$$

$$\left( \begin{matrix} \begin{matrix} b_{11} & b_{12} & b_{13} \\ & b_{22} & b_{21} \\ & & b_{33} \end{matrix} & \begin{matrix} & & \\ b_{23} & & \\ b_{34} & b_{35} & \end{matrix} & \begin{matrix} & & \\ & & \\ & & \end{matrix} \\ \begin{matrix} & & \\ & & \\ & & \end{matrix} & \begin{matrix} & & \\ & & \\ & & \end{matrix} & \begin{matrix} & & \\ & & \\ & & \end{matrix} \\ \begin{matrix} & & \\ & & \\ & & \end{matrix} & \begin{matrix} & b_{(N-4)(N-4)} & b_{(N-4)(N-3)} \\ & & b_{(N-3)(N-3)} \\ & & \end{matrix} & \begin{matrix} b_{(N-4)N-2)} & & \\ b_{(N-3)(N-2)} & b_{(N-3)(N-1)} & \\ b_{(N-2)(N-2)} & b_{(N-2)(N-1)} & b_{(N-2)(N)} \end{matrix} \end{matrix} \right)_{(N-2)\times N}\left( \begin{matrix} \begin{matrix} \mu_{1} \\ \mu_{2} \\ \mu_{3} \end{matrix} \\ \begin{matrix} \vdots\\ \vdots\\ \vdots\end{matrix} \\ \begin{matrix} \mu_{N-2} \\ \mu_{N-1} \\ \mu_{N} \end{matrix} \end{matrix} \right)$$

Matrix Representation of Eq. 39 :

| $\left[ A^{*} \right]_{\left( N-2 \right)\times(N-2)}{[Z^{*}]}_{(N-2)\times1}=\left[ B^{*} \right]_{\left( N-2 \right)\times(N-2)}{[\mu^{*}]}_{(N-2)\times1}$  $\left( \begin{matrix} \begin{matrix} a_{12} & a_{13} \\ a_{22} & a_{21} \\ & a_{33} \end{matrix} & \begin{matrix} & & \\ a_{23} & & \\ a_{34} & a_{35} & \end{matrix} & \begin{matrix} & \\ & \\ & \end{matrix} \\ \begin{matrix} & \\ & \\ & \end{matrix} & \begin{matrix} & & \\ & & \\ & & \end{matrix} & \begin{matrix} & \\ & \\ & \end{matrix} \\ \begin{matrix} & \\ & \\ & \end{matrix} & \begin{matrix} & a_{(N-4)(N-4)} & a_{(N-4)(N-3)} \\ & & a_{(N-3)(N-3)} \\ & & \end{matrix} & \begin{matrix} a_{(N-4)(N-2)} & \\ a_{(N-3)(N-2)} & a_{(N-3)(N-1)} \\ a_{(N-2)(N-2)} & a_{(N-2)(N-1)} \end{matrix} \end{matrix} \right)_{(N-2)\times(N-2)}\left( \begin{matrix} \begin{matrix} Z_{2} \\ Z_{3} \end{matrix} \\ \begin{matrix} \vdots\\ \vdots\\ \vdots\end{matrix} \\ \begin{matrix} Z_{N-2} \\ Z_{N-1} \end{matrix} \end{matrix} \right)=\left( \begin{matrix} \begin{matrix} b_{12} & b_{13} \\ b_{22} & b_{21} \\ & b_{33} \end{matrix} & \begin{matrix} & & \\ b_{23} & & \\ b_{34} & b_{35} & \end{matrix} & \begin{matrix} & \\ & \\ & \end{matrix} \\ \begin{matrix} & \\ & \\ & \end{matrix} & \begin{matrix} & & \\ & & \\ & & \end{matrix} & \begin{matrix} & \\ & \\ & \end{matrix} \\ \begin{matrix} & \\ & \\ & \end{matrix} & \begin{matrix} & b_{(N-4)(N-4)} & b_{(N-4)(N-3)} \\ & & b_{(N-3)(N-3)} \\ & & \end{matrix} & \begin{matrix} b_{(N-4)(N-2)} & \\ b_{(N-3)(N-2)} & b_{(N-3)(N-1)} \\ b_{(N-2)(N-2)} & b_{(N-2)(N-1)} \end{matrix} \end{matrix} \right)_{(N-2)\times(N-2)}\left( \begin{matrix} \begin{matrix} \mu_{2} \\ \mu_{3} \end{matrix} \\ \begin{matrix} \vdots\\ \vdots\\ \vdots\end{matrix} \\ \begin{matrix} \mu_{N-2} \\ \mu_{N-1} \end{matrix} \end{matrix} \right)$ | (39) |
| --- | --- |

Matrix Representation of Eq. 40 :

| $\left[ C \right]_{(N-2)\times N}{[Z]}_{N\times1}+\left[ D \right]_{(N-2)\times N}{[\mu]}_{N\times1}=\left[ K \right]_{(N-2)\times N}{[\varphi]}_{N\times1}$  $\left( \begin{matrix} \begin{matrix} c_{11} & c_{12} & c_{13} \\ & c_{22} & c_{21} \\ & & c_{33} \end{matrix} & \begin{matrix} & & \\ c_{23} & & \\ c_{34} & c_{35} & \end{matrix} & \begin{matrix} & & \\ & & \\ & & \end{matrix} \\ \begin{matrix} & & \\ & & \\ & & \end{matrix} & \begin{matrix} & & \\ & & \\ & & \end{matrix} & \begin{matrix} & & \\ & & \\ & & \end{matrix} \\ \begin{matrix} & & \\ & & \\ & & \end{matrix} & \begin{matrix} & c_{(N-4)(N-4)} & c_{(N-4)(N-3)} \\ & & c_{(N-3)(N-3)} \\ & & \end{matrix} & \begin{matrix} c_{(N-4)(N-2)} & & \\ c_{(N-3)(N-2)} & c_{(N-3)(N-1)} & \\ c_{(N-2)(N-2)} & c_{(N-2)(N-1)} & c_{(N-2)(N)} \end{matrix} \end{matrix} \right)_{(N-2)\times N}\left( \begin{matrix} \begin{matrix} Z_{1} \\ Z_{2} \\ Z_{3} \end{matrix} \\ \begin{matrix} \vdots\\ \vdots\\ \vdots\end{matrix} \\ \begin{matrix} Z_{N-2} \\ Z_{N-1} \\ Z_{N} \end{matrix} \end{matrix} \right)+\left( \begin{matrix} \begin{matrix} d_{11} & d_{12} & d_{13} \\ & d_{22} & d_{21} \\ & & d_{33} \end{matrix} & \begin{matrix} & & \\ d_{23} & & \\ d_{34} & d_{35} & \end{matrix} & \begin{matrix} & & \\ & & \\ & & \end{matrix} \\ \begin{matrix} & & \\ & & \\ & & \end{matrix} & \begin{matrix} & & \\ & & \\ & & \end{matrix} & \begin{matrix} & & \\ & & \\ & & \end{matrix} \\ \begin{matrix} & & \\ & & \\ & & \end{matrix} & \begin{matrix} & d_{(N-4)(N-4)} & d_{(N-4)(N-3)} \\ & & d_{(N-3)(N-3)} \\ & & \end{matrix} & \begin{matrix} d_{(N-4)(N-2)} & & \\ d_{(N-3)(N-2)} & d_{(N-3)(N-1)} & \\ d_{(N-2)(N-2)} & d_{(N-2)(N-1)} & d_{(N-2)(N)} \end{matrix} \end{matrix} \right)_{(N-2)\times N}\left( \begin{matrix} \begin{matrix} \mu_{1} \\ \mu_{2} \\ \mu_{3} \end{matrix} \\ \begin{matrix} \vdots\\ \vdots\\ \vdots\end{matrix} \\ \begin{matrix} \mu_{N-2} \\ \mu_{N-1} \\ \mu_{N} \end{matrix} \end{matrix} \right)=\left( \begin{matrix} \begin{matrix} k_{11} & k_{12} & k_{13} \\ & k_{22} & k_{21} \\ & & k_{33} \end{matrix} & \begin{matrix} & & \\ k_{23} & & \\ k_{34} & k_{35} & \end{matrix} & \begin{matrix} & & \\ & & \\ & & \end{matrix} \\ \begin{matrix} & & \\ & & \\ & & \end{matrix} & \begin{matrix} & & \\ & & \\ & & \end{matrix} & \begin{matrix} & & \\ & & \\ & & \end{matrix} \\ \begin{matrix} & & \\ & & \\ & & \end{matrix} & \begin{matrix} & k_{(N-4)(N-4)} & k_{(N-4)(N-3)} \\ & & k_{(N-3)(N-3)} \\ & & \end{matrix} & \begin{matrix} k_{(N-4)(N-2)} & & \\ k_{(N-3)(N-2)} & k_{(N-3)(N-1)} & \\ k_{(N-2)(N-2)} & k_{(N-2)(N-1)} & k_{(N-2)(N)} \end{matrix} \end{matrix} \right)_{(N-2)\times N}\left( \begin{matrix} \begin{matrix} \varphi_{1} \\ \varphi_{2} \\ \varphi_{3} \end{matrix} \\ \begin{matrix} \vdots\\ \vdots\\ \vdots\end{matrix} \\ \begin{matrix} \varphi_{N-2} \\ \varphi_{N-1} \\ \varphi_{N} \end{matrix} \end{matrix} \right)$ | (40) |
| --- | --- |

Matrix Representation of Eq. 41 :

| $\left[ C^{*} \right]_{\left( N-2 \right)\times(N-2)}{[Z^{*}]}_{(N-2)\times1}+\left[ D^{*} \right]_{\left( N-2 \right)\times(N-2)}{[\mu^{*}]}_{(N-2)\times1}=\left[ K^{*} \right]_{\left( N-2 \right)\times(N-2)}{[\varphi^{*}]}_{(N-2)\times1}+{[U_{0}]}_{(N-2)\times1}$  $\left( \begin{matrix} \begin{matrix} c_{12} & c_{13} \\ c_{22} & c_{21} \\ & c_{33} \end{matrix} & \begin{matrix} & & \\ c_{23} & & \\ c_{34} & c_{35} & \end{matrix} & \begin{matrix} & \\ & \\ & \end{matrix} \\ \begin{matrix} & \\ & \\ & \end{matrix} & \begin{matrix} & & \\ & & \\ & & \end{matrix} & \begin{matrix} & \\ & \\ & \end{matrix} \\ \begin{matrix} & \\ & \\ & \end{matrix} & \begin{matrix} & c_{(N-4)(N-4)} & c_{(N-4)(N-3)} \\ & & a_{(N-3)(N-3)} \\ & & \end{matrix} & \begin{matrix} c_{(N-4)(N-2)} & \\ c_{(N-3)(N-2)} & c_{(N-3)(N-1)} \\ c_{(N-2)(N-2)} & c_{(N-2)(N-1)} \end{matrix} \end{matrix} \right)_{(N-2)\times(N-2)}\left( \begin{matrix} \begin{matrix} Z_{2} \\ Z_{3} \end{matrix} \\ \begin{matrix} \vdots\\ \vdots\\ \vdots\end{matrix} \\ \begin{matrix} Z_{N-2} \\ Z_{N-1} \end{matrix} \end{matrix} \right)+\left( \begin{matrix} \begin{matrix} d_{12} & d_{13} \\ d_{22} & d_{21} \\ & d_{33} \end{matrix} & \begin{matrix} & & \\ d_{23} & & \\ d_{34} & d_{35} & \end{matrix} & \begin{matrix} & \\ & \\ & \end{matrix} \\ \begin{matrix} & \\ & \\ & \end{matrix} & \begin{matrix} & & \\ & & \\ & & \end{matrix} & \begin{matrix} & \\ & \\ & \end{matrix} \\ \begin{matrix} & \\ & \\ & \end{matrix} & \begin{matrix} & d_{(N-4)(N-4)} & d_{(N-4)(N-3)} \\ & & d_{(N-3)(N-3)} \\ & & \end{matrix} & \begin{matrix} d_{(N-4)(N-2)} & \\ d_{(N-3)(N-2)} & d_{(N-3)(N-1)} \\ d_{(N-2)(N-2)} & d_{(N-2)(N-1)} \end{matrix} \end{matrix} \right)_{(N-2)\times(N-2)}\left( \begin{matrix} \begin{matrix} \mu_{2} \\ \mu_{3} \end{matrix} \\ \begin{matrix} \vdots\\ \vdots\\ \vdots\end{matrix} \\ \begin{matrix} \mu_{N-2} \\ \mu_{N-1} \end{matrix} \end{matrix} \right)$  $=\left( \begin{matrix} \begin{matrix} k_{12} & k_{13} \\ k_{22} & k_{21} \\ & k_{33} \end{matrix} & \begin{matrix} & & \\ k_{23} & & \\ k_{34} & k_{35} & \end{matrix} & \begin{matrix} & \\ & \\ & \end{matrix} \\ \begin{matrix} & \\ & \\ & \end{matrix} & \begin{matrix} & & \\ & & \\ & & \end{matrix} & \begin{matrix} & \\ & \\ & \end{matrix} \\ \begin{matrix} & \\ & \\ & \end{matrix} & \begin{matrix} & k_{(N-4)(N-4)} & k_{(N-4)(N-3)} \\ & & k_{N-3N-3} \\ & & \end{matrix} & \begin{matrix} k_{(N-4)(N-2)} & \\ k_{(N-3)(N-2)} & k_{(N-3)(N-1)} \\ k_{(N-2)(N-2)} & k_{(N-2)(N-1)} \end{matrix} \end{matrix} \right)_{(N-2)\times(N-2)}\left( \begin{matrix} \begin{matrix} \varphi_{2} \\ \varphi_{3} \end{matrix} \\ \begin{matrix} \vdots\\ \vdots\\ \vdots\end{matrix} \\ \begin{matrix} \varphi_{N-2} \\ \varphi_{N-1} \end{matrix} \end{matrix} \right)+\left( \begin{matrix} \begin{matrix} {k_{11}\varphi}_{1} \\ 0 \end{matrix} \\ \begin{matrix} \vdots\\ \vdots\\ \vdots\end{matrix} \\ \begin{matrix} 0 \\ {k_{(N-2)(N)}\varphi}_{N} \end{matrix} \end{matrix} \right)$ | (41) |
| --- | --- |

Matrix Representation of Eq. 42 :

$$\left( \begin{matrix} \begin{matrix} V_{2} \\ V_{3} \end{matrix} \\ \begin{matrix} \vdots\\ \vdots\\ \vdots\end{matrix} \\ \begin{matrix} V_{N-2} \\ V_{N-1} \end{matrix} \end{matrix} \right)=\left( \begin{matrix} \begin{matrix} \varphi_{2} \\ \varphi_{3} \end{matrix} \\ \begin{matrix} \vdots\\ \vdots\\ \vdots\end{matrix} \\ \begin{matrix} \varphi_{N-2} \\ \varphi_{N-1} \end{matrix} \end{matrix} \right)-\frac{1}{4}{\left( \begin{matrix} \begin{matrix} r_{2} & \\ & r_{3} \end{matrix} & \begin{matrix} & \\ & \end{matrix} & \begin{matrix} & \\ & \end{matrix} \\ \begin{matrix} & \\ & \end{matrix} & \begin{matrix} \ddots& \\ & \ddots\end{matrix} & \begin{matrix} & \\ & \end{matrix} \\ \begin{matrix} & \\ & \end{matrix} & \begin{matrix} & \\ & \end{matrix} & \begin{matrix} r_{N-2} & \\ & r_{N-1} \end{matrix} \end{matrix} \right)^{2}}_{(N-2)\times(N-2)}\left( \begin{matrix} \begin{matrix} \mu_{2} \\ \mu_{3} \end{matrix} \\ \begin{matrix} \vdots\\ \vdots\\ \vdots\end{matrix} \\ \begin{matrix} \mu_{N-2} \\ \mu_{N-1} \end{matrix} \end{matrix} \right)$$

$$+\frac{1}{64}{\left( \begin{matrix} \begin{matrix} r_{2} & \\ & r_{3} \end{matrix} & \begin{matrix} & \\ & \end{matrix} & \begin{matrix} & \\ & \end{matrix} \\ \begin{matrix} & \\ & \end{matrix} & \begin{matrix} \ddots& \\ & \ddots\end{matrix} & \begin{matrix} & \\ & \end{matrix} \\ \begin{matrix} & \\ & \end{matrix} & \begin{matrix} & \\ & \end{matrix} & \begin{matrix} r_{N-2} & \\ & r_{N-1} \end{matrix} \end{matrix} \right)^{4}}_{(N-2)\times(N-2)}\left( \begin{matrix} \begin{matrix} Z_{2} \\ Z_{3} \end{matrix} \\ \begin{matrix} \vdots\\ \vdots\\ \vdots\end{matrix} \\ \begin{matrix} Z_{N-2} \\ Z_{N-1} \end{matrix} \end{matrix} \right)$$
